# CEEMDAN-decomposed time series forecasting of reported hepatitis B cases using KOA-optimised deep learning: a nationwide study in mainland China (2004–2027)

**DOI:** 10.7189/jogh.16.04200

**Published:** 2026-09-18

**Authors:** Zhende Wang, Fengying Bi, Zhichao Duan, Li Ding, Bin Song, Ke Wang, Jinyu Zhao, Haiying Li

**Affiliations:** Department of Human Resource, Zibo Central Hospital, Zibo, China

**Keywords:** hepatitis B, epidemiology, modelling, prediction

## Abstract

**Background:**

Hepatitis B remains a leading notifiable infection in mainland China, with a persistent burden shaped by chronic reservoirs, varied immunity, and shifting surveillance practices. Reliable short- to medium-term forecasts of reported hepatitis B cases are therefore valuable for planning diagnostics, care pathways, antiviral supply, and targeted prevention.

**Methods:**

We compiled a national monthly series of hepatitis B notifications from January 2004 to December 2025 and applied Complete Ensemble Empirical Mode Decomposition with Adaptive Noise (CEEMDAN) to isolate multiscale temporal components. Four modelling approaches – gated recurrent units (GRU), convolutional neural networks (CNN), support vector machines (SVM), and a Transformer encoder – were trained on CEEMDAN-derived features using a sliding 12-month window and recursively extended to 24-month horizons. Hyperparameters were optimised *via* the Kepler Optimization Algorithm (KOA), while performance was assessed through R^2^, Root Mean Square Error (RMSE), Mean Absolute Error (MAE), Mean Absolute Percentage Error (MAPE), and regression diagnostics across training, validation, and test splits.

**Results:**

All models captured dominant trends and seasonality; on the held-out test split, Transformer again delivered the best out-of-sample fit (MAE = 3105.508, MAPE = 0.024, RMSE = 4071.901, and R^2^ = 0.928), while SVM ranked second with MAE, MAPE and RMSE values that were 11.317%, 11.615%, and 9.964% higher than the Transformer model’s. CNN performed better than GRU but worse than SVM on the test set, achieving MAE, MAPE and RMSE reductions of 21.985%, 22.178%, and 14.944% relative to GRU, alongside a 6.158% larger R^2^. Forecasts for 2026–2027 remain elevated, signalling little improvement and even possible resurgence.

**Conclusions:**

The findings demonstrate that integrating CEEMDAN with KOA optimised machine learning models delivers highly reliable short-term forecasts. This hybrid framework establishes a resilient continuum from epidemiological surveillance to predictive modelling and public health decision making, thereby providing essential foresight to guide optimal resource allocation and proactive intervention strategies for Hepatitis B control.

Hepatitis B continues to pose a substantial global health burden due to its high prevalence, chronicity, and potential for serious liver-related complications [1–4]. In March 2015, the World Health Organization (WHO) released the first comprehensive guidelines for the prevention, diagnosis, care and treatment of chronic hepatitis B framing a global elimination agenda that aims to reduce incidence by 90% and mortality by 65% by 2030 [5,6]. Until today, most countries remain off track, with the situation especially urgent in low and middle-income countries [4,7]. This burden is particularly pronounced in mainland China, which is home to approximately one-third of the world’s hepatitis B virus (HBV) infection [8,9]. Although universal vaccination against HBV began in mainland China in 1992 and has substantially lowered transmission and mortality, modelling studies still project around 60 million people living with HBV and 680,000 deaths by 2030 [10]. For these reasons, reliable short-term and medium-term forecasts of notified cases are valuable for public-health authorities [11,12].

Time-series modelling by machine learning and deep learning techniques have increasingly been applied to infectious-disease forecasting, for their ability to learn complex representations directly from data. Despite this progress, few studies have explored large language model-inspired or attention-based approaches in the specific context of hepatitis B, leaving a gap in the evidence base for how these advanced architectures perform relative to more conventional frameworks.

Infectious disease series often mix long-term trends, seasonal oscillations, and irregular components [13], so direct modelling without prior decomposition may introduce instability or reduce forecast accuracy. Moreover, the growing interest in hyperparameter optimisation is closely tied to the pursuit of creating reliable deep learning models for numerous applications [14], since many existing modelling efforts rely on empirical or exhaustive hyperparameter tuning, which can be time-consuming and inefficient [15].

Improving modelling efficiency and fidelity has tangible public health value. More accurate and interpretable forecasts can enhance the monitoring-prediction-decision pathway by providing timely intelligence for surveillance, guiding resource allocation, and supporting proactive interventions. Integrating robust forecasting tools into routine practice can help health systems respond more effectively to the persistent challenge of hepatitis B and contribute to the long-term goal of reducing its burden.

## METHODS

### Data sources

Data on the monthly number of reported hepatitis B cases in mainland China from January 2004 to December 2025, comprising a total of 264 months, were extracted from the Monthly Overview of Notifiable Infectious Disease. To ensure data completeness and continuity, we established a strict hierarchical retrieval process. The official website of the National Disease Control and Prevention Administration served as our absolute primary data source. When specific historical data points were inaccessible on this platform, we queried the official website of the Chinese Center for Disease Control and Prevention as our primary supplementary channel. Only when data remained unavailable from both official websites did we consult secondary reproduced reports published on the China National Knowledge Infrastructure. The complete original data set extracted for this study is available in Table S1 in the **Online Supplementary Document**. We strictly adhered to the GRABDROP guidelines for secondary data analyses [16]. Detailed explanations regarding our adherence are provided in Table S1 in the **Online Supplementary Document**.

### Sample construction and preprocessing

Supervised samples were constructed with a fixed 12-month look-back and a one-step-ahead target, sliding by one month. Because the first 12 months were consumed in forming the initial window, they yielded no targets; the data set therefore provided 252 supervised outputs and 252 corresponding inputs. Chronological splits allocated 202 samples to training (80%), 25 to validation (10%), and 25 to testing (10%). Hyperparameter selection and model choice were based exclusively on validation performance; the test split was held out for final evaluation.

All inputs were standardised using z-score normalisation. Crucially, to ensure zero data leakage, the normalisation parameters were estimated using only the training data and then applied to the validation and testing data sets. Predictions were inverse-transformed to the original scale prior to evaluation.

### CEEMDAN decomposition procedure

We decomposed the original monthly reported hepatitis B cases series by CEEMDAN, implemented following the original algorithm of Torres *et al.* [17–21]. More detailed parameter settings are provided in Parameter settings and data decomposition results based on CEEMDAN in the **Online Supplementary Document**.

Following the CEEMDAN decomposition, a Decomposition-Prediction-Reconstruction strategy was adopted. Specifically, the prediction models were trained to forecast each individual intrinsic mode function (IMF) and the residual series, the final fitted notified cases was reconstructed by linearly summing the individual forecasted IMFs and the forecasted residual.

### Model architectures and training regime

GRU architecture mirrors stacked Long Short-Term Memory (LSTM) depth but uses two gated recurrent layers each followed by dropout, feeding a fully connected layer before regression output. CNN employs convolutional filters along the time axis, batch normalisation, max-pooling repeated twice, dropout, and flattening prior to fully connected and regression layers. Transformer encoder embeds inputs, adds positional encodings, processes embeddings *via* multi-head self-attention with dropout repeated twice, then fully connected and regression layers. SVM uses lagged observations as fixed-dimensional inputs, a radial basis function kernel, epsilon-insensitive loss, and tuned kernel scale, box constraint, and epsilon for CEEMDAN components. Training uses Adam, batch size 20, maximum 500 epochs, fixed seeds, early stopping on validation loss, inputs scaled using training statistics, and outputs inverse-transformed before metric computation (Figure S1 in the **Online Supplementary Document**).

### Hyperparameter optimisation by KOA

Model hyperparameters were optimised using the KOA, a population-based, physics-inspired metaheuristic that treats each candidate hyperparameter vector as a ‘planet’ and the best solution found to date as the ‘Sun’ [22]. A population of candidate solutions is initialised uniformly within prescribed bounds and each candidate is assigned random orbital attributes to increase diversity. Candidates are evaluated using the validation RMSE and the best evaluated solution is recorded as the Sun. The optimal configurations identified by KOA on the validation set were then fixed and used for the final training and evaluation on the held-out test set. The source code of KOA is publicly accessible [22]. The settings of KOA and the search ranges and set values of all model parameters are presented in Table S2 in the **Online Supplementary Document**.

### Goodness-of-fit diagnosis

Evaluation uses R^2^, RMSE, MAE, and MAPE computed on the training, validation, and test splits. In addition to error metrics, linear diagnostics are conducted by regressing observed values on predictions for each model and split; slope, intercept, and coefficient of determination (R^2^) are reported to assess calibration and scale alignment.



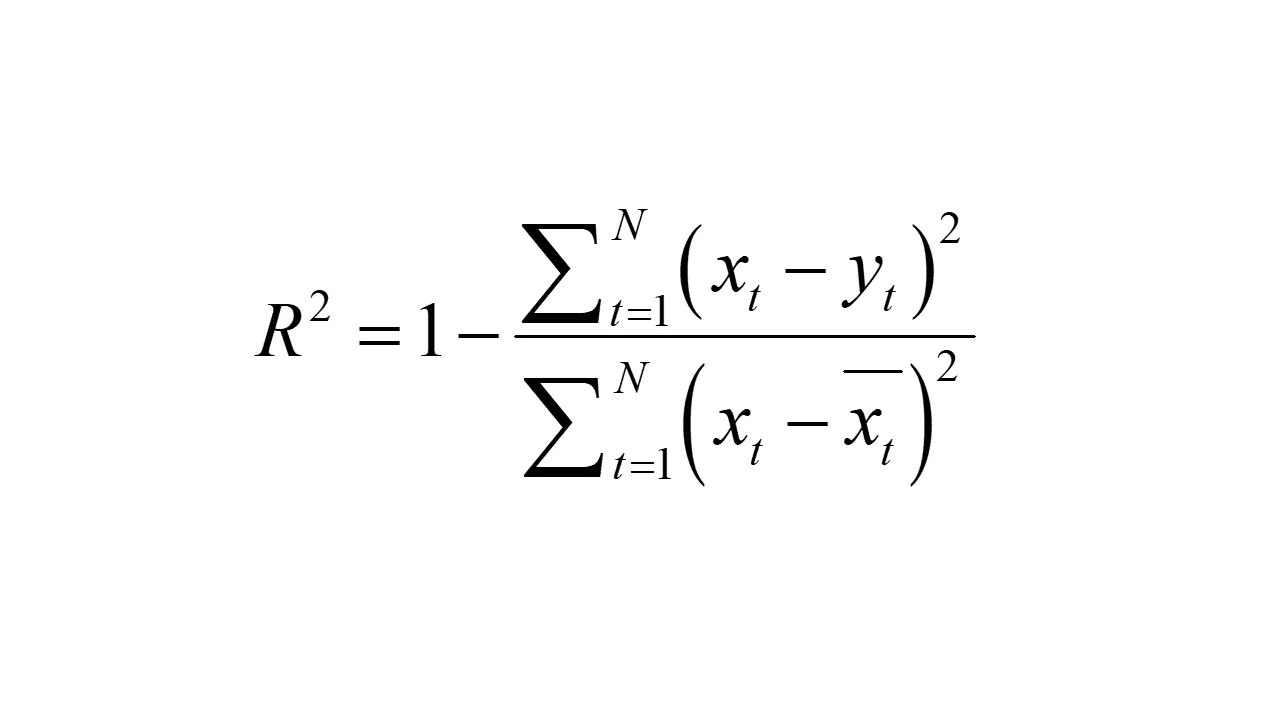





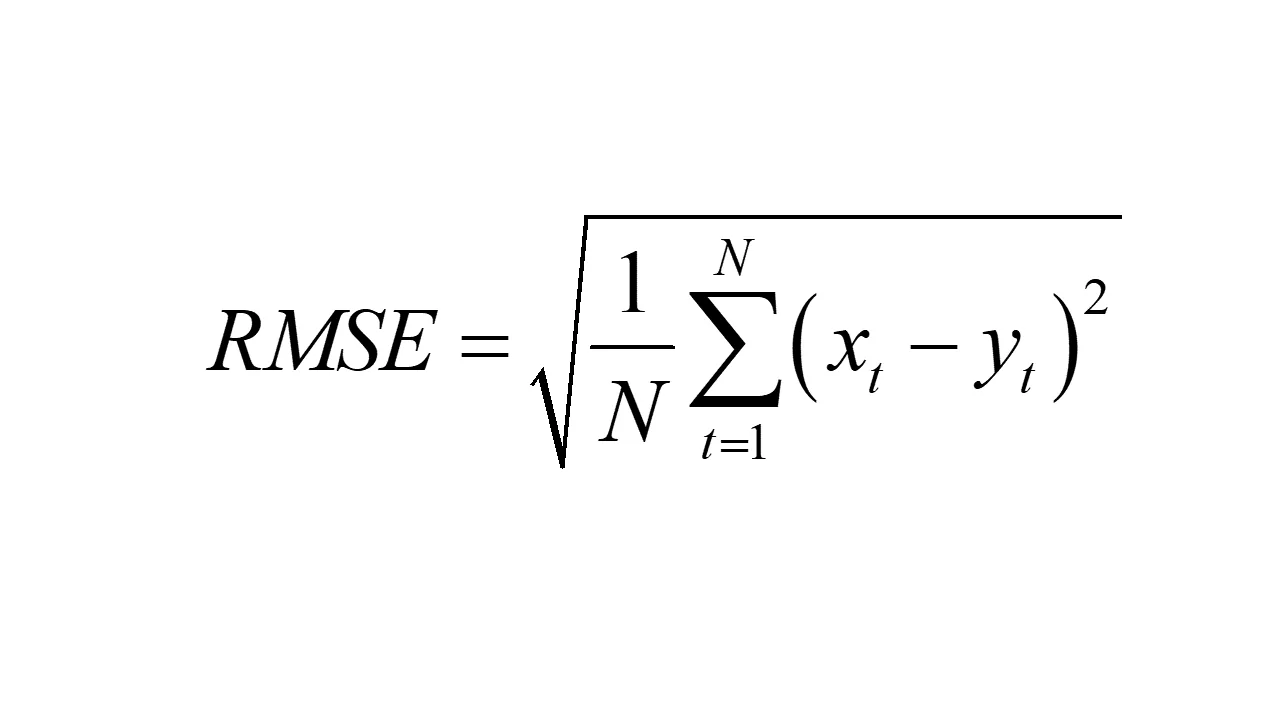





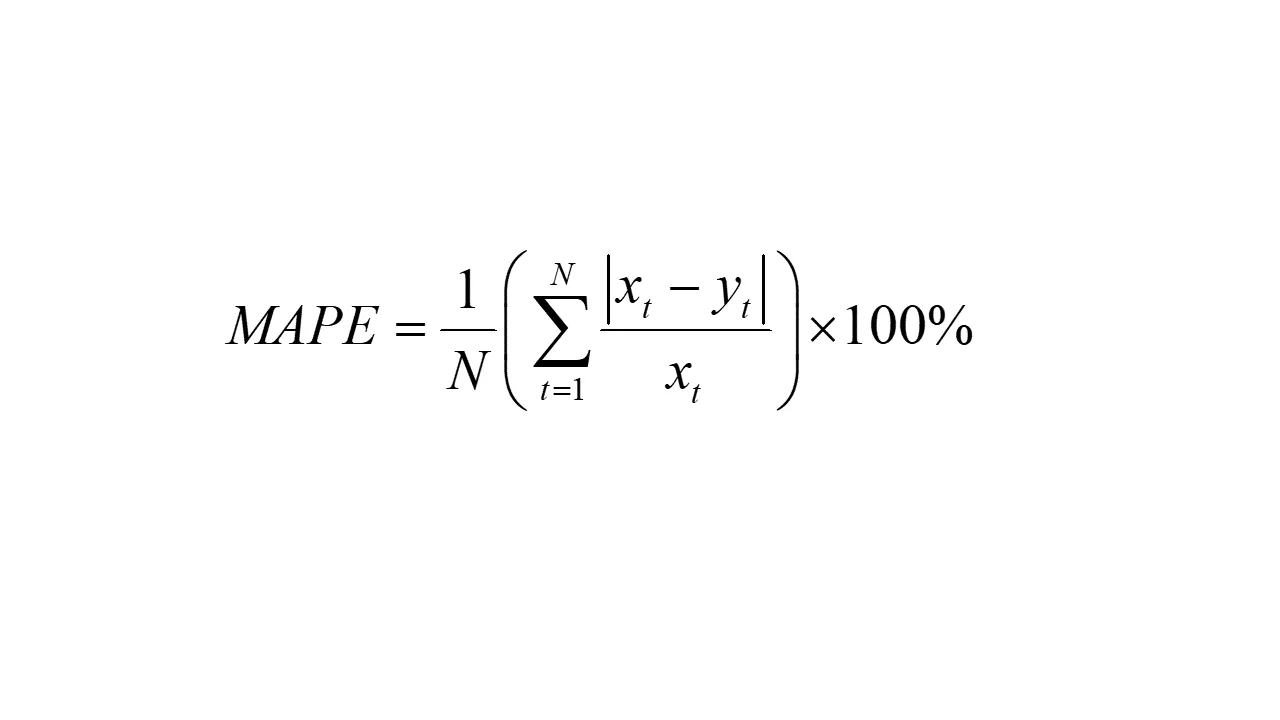





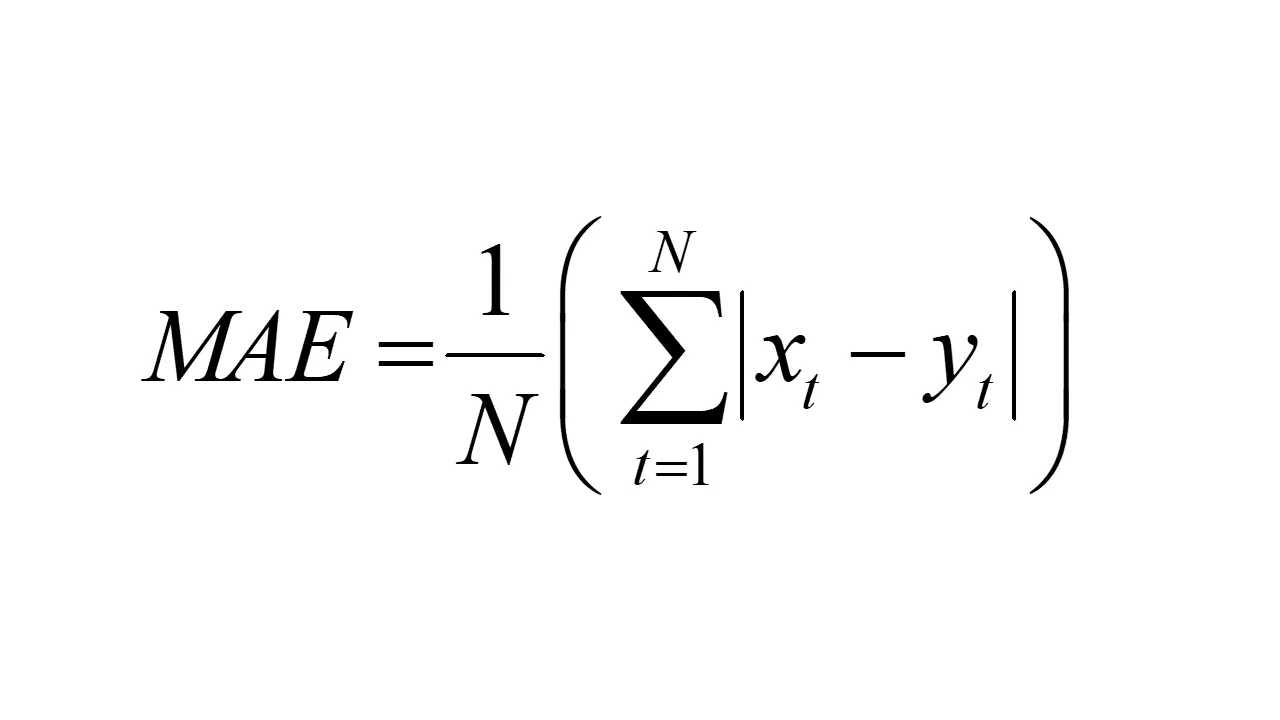



Where *x_t_* and *y_t_* denote the original and simulated series, respectively.

We conducted a comprehensive residual analysis to evaluate the model calibration. The autocorrelation function of the prediction errors was computed to identify any remaining seasonal error structures or unexplained temporal dependencies. Furthermore, we systematically analysed the temporal distribution of the model residuals across the historical timeline to determine whether significant deviations clustered around specific socio behavioural anomalies or pandemic disruptions.

### Sensitivity analysis

To rigorously test the robustness of our predictive architectures and mitigate the disproportionate influence of extreme pandemic era outliers, we conducted a formal sensitivity analysis. The anomalous data points corresponding to the initial COVID-19 outbreak (February 2020) and the subsequent major healthcare policy transition (December 2022) were systematically removed from the original data set. To reconstruct a continuous sequence without distorting the underlying cyclical patterns, we applied cubic spline interpolation to estimate the baseline reporting volume for these specific missing months. All predictive models were subsequently retrained and evaluated on this interpolated data set to assess their structural stability.

### Probabilistic interval prediction

To quantify forecasting uncertainty while accommodating the potential asymmetry and non-normality of epidemiological data, non-parametric Kernel Density Estimation (KDE) was employed to construct prediction intervals. Specifically, prediction errors (residuals) were first computed from the validation set. An Epanechnikov kernel function was applied to fit the empirical probability density function and the corresponding cumulative distribution function (CDF) of these validation errors. For target confidence levels of 0.95, 0.65, and 0.35, the lower and upper error bounds were directly extracted from specific percentiles of the KDE-derived CDF. Subsequently, these data-driven, potentially asymmetric error bounds were superimposed onto the point forecasts to formulate the final prediction intervals for the recursive 24-month future horizon.

### Software for modelling

Data collection and organisation were completed based on the Microsoft Office 2024 software. All operations for modelling in the research were carried out under the MATLAB R2024a software (The MathWorks, Inc., Natick, MA, USA).

## RESULTS

### Characteristics of the time series

**Figure 1:**
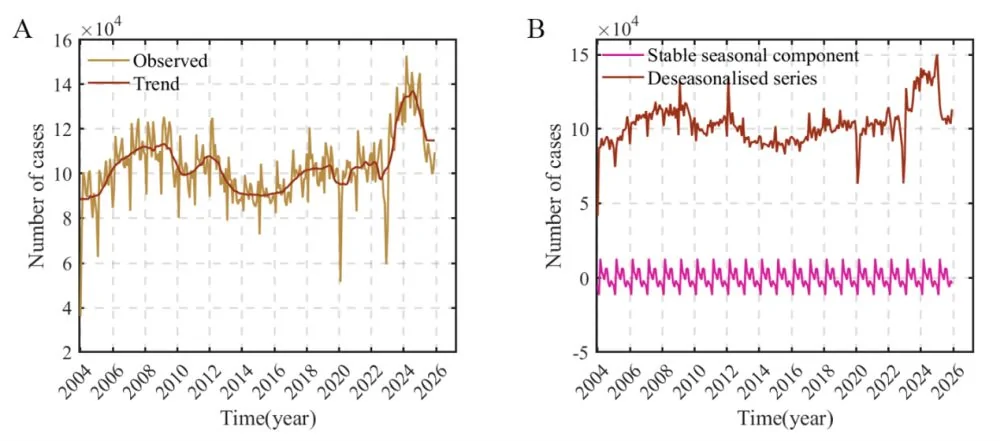
The long-term trend and seasonal fluctuations of the reported hepatitis B cases.

During the study period, the temporal trend of notified hepatitis B cases in mainland China displayed a multi-phasic rise-fall-rise pattern with stable periodic fluctuations (**Figure 1**, Panel A). Following an initial surge, monthly notifications fluctuated primarily between 80,000 and 130,000, characterised by pronounced spring and autumn peaks during the 2006–2009 high-reporting period. Between 2010 and 2016, the volume of reported cases stabilised at an average of 96,279, though the recurrent March spikes persisted. A subsequent rebound in the late 2010s was marked by strong spring peaks and elevated summer counts, which were abruptly interrupted by deep, pandemic-related troughs in February 2020 (51,506) and December 2022 (59,498). From 2023 onward, monthly values repeatedly surpassed 120,000, culminating in a historical maximum of 152,967 in March 2024. The underlying seasonal component exhibited a intra-annual pattern, featuring pronounced negative deviations in February, October, and December, contrasting with prominent positive peaks in March, July, and August (**Figure 1**, Panel B).

### CEEMDAN data decomposition results

Prior to predictive modelling, the CEEMDAN algorithm decomposed the monthly hepatitis B notification series into seven intrinsic mode functions (IMF1–IMF7) and one residual component, ordered from highest to lowest characteristic frequency (Figure S2 and Table S3 in the **Online Supplementary Document**).

### The fitting effects of the models

**Figure 2:**
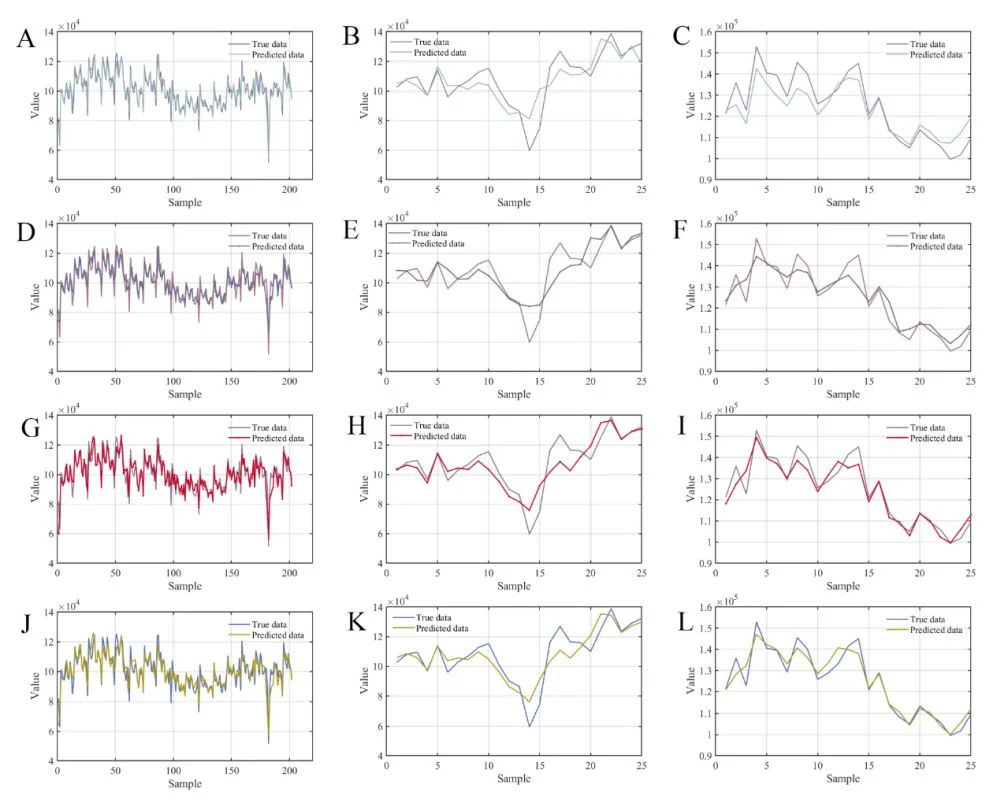
The fitting effects of all models. Panels illustrate the alignment between the monthly notified cases and the fitted values generated by the four models across the training, validation, and test partitions. Panels A–C. The GRU. Panels D–F. The CNN. Panels G–I. The SVM. Panels J–L. The Transformer. CNN – Convolutional Neural Network, GRU – Gated Recurrent Unit, SVM – Support Vector Machine.

All models captured the underlying temporal dynamics, yielding fitting curves that closely matches the original data (**Figure 2**) and high training R^2^ values (GRU = 0.902, CNN = 0.740, SVM = 0.773, Transformer = 0.776). Evaluation on the test set confirmed robust out-of-sample forecasting, with the Transformer demonstrating superior generalisation (Test R^2^ = 0.93) (Figures S3 and S4 and Table S4 in the **Online Supplementary Document**).

Evaluation of the residual autocorrelation function revealed no significant periodic spikes in the residual sequence, indicating that all models effectively captured the predominant cyclical structures. However, a temporal sequence analysis demonstrated that the forecast errors were not completely randomly distributed. Instead, the most pronounced residual deviations consistently clustered around major calendar anomalies. Specifically, predictive errors were noticeably magnified during the Spring Festival months of January and February. Similarly, structural error clusters were identified during the initial outbreak of the COVID-19 pandemic in early 2020 and the subsequent policy transition period in late 2022 (Figure S5 and S6 in the **Online Supplementary Document**).

### Goodness-of-fit across training, validation, and test splits

The GRU achieved the most accurate fit on the training set (MAE = 2679.238, MAPE = 0.027, RMSE = 3549.723, R^2^ = 0.902), this in-sample superiority failed to generalise to the hold-out set. On the validation set, the Transformer yielded the best performance (R^2^ = 0.792, RMSE = 8060.361), closely followed by the SVM, whose metrics deviated by less than 6.3% across all indicators. This advantage was further solidified on the test set, where the Transformer achieved the highest predictive accuracy (MAE = 3105.508, MAPE = 0.024, RMSE = 4071.901, R^2^ = 0.928). Furthermore, the CNN consistently outperformed the GRU on the test set by demonstrating substantial error reductions and the larger R^2^ (0.868) (**Table 1**). The results of the sensitivity analysis demonstrated robust consistency with the findings derived from the original data set (Figures S7–9 in the **Online Supplementary Document**). Evaluation of the models on the interpolated data revealed that the Transformer architecture retained its superior generalisation capabilities on the test set (MAE = 3044.484, MAPE = 0.024, RMSE = 4209.168, R^2^ = 0.923) (Table S5 in the **Online Supplementary Document**).

**Table 1:** Comparative goodness-of-fit metrics for all models on train, validation, and test sets

Models	Training set fitting power				Validation set fitting power				Test set fitting power			
	**MAE**	**MAPE**	**RMSE**	**R^2^**	**MAE**	**MAPE**	**RMSE**	**R^2^**	**MAE**	**MAPE**	**RMSE**	**R^2^**
GRU	2,679.238	0.027	3,549.723	0.902	7,304.553	0.076	9,664.264	0.701	5,356.391	0.042	6,494.535	0.818
CNN	4,181.923	0.043	5,791.381	0.739	6,677.803	0.069	9,759.442	0.695	4,178.806	0.033	5,524.005	0.868
SVM	3,882.565	0.040	5,433.854	0.771	6,450.456	0.066	8,597.948	0.763	3,501.811	0.027	4,522.501	0.912
Transformer	4,006.670	0.041	5,379.282	0.775	6,165.475	0.063	8,060.361	0.792	3,105.508	0.024	4,071.901	0.928
GRU *vs.* CNN	56.086%	60.499%	63.150%	18.033%	8.580%	8.603%	0.985%	0.844%	21.985%	22.178%	14.944%	6.158%
GRU *vs.* SVM	44.913%	46.946%	53.078%	14.577%	11.693%	13.139%	11.034%	8.890%	34.624%	35.705%	30.365%	11.470%
GRU *vs.* Transformer	49.545%	50.551%	51.541%	14.069%	15.594%	16.942%	16.596%	12.978%	42.022%	43.173%	37.303%	13.515%
CNN *vs.* SVM	7.158%	8.444%	6.173%	4.217%	3.405%	4.963%	11.901%	9.817%	16.201%	17.382%	18.130%	5.004%
CNN *vs.* Transformer	4.191%	6.198%	7.116%	4.837%	7.672%	9.124%	17.410%	13.940%	25.684%	26.978%	26.287%	6.930%
SVM *vs.* Transformer	3.197%	2.454%	1.004%	0.595%	4.418%	4.378%	6.252%	3.754%	11.317%	11.615%	9.964%	1.834%

MAE – Mean Absolute Error, MAPE – Mean Absolute Percentage Error, RMSE – Root Mean Square Error

### The prediction results of the models

**Figure 3:**
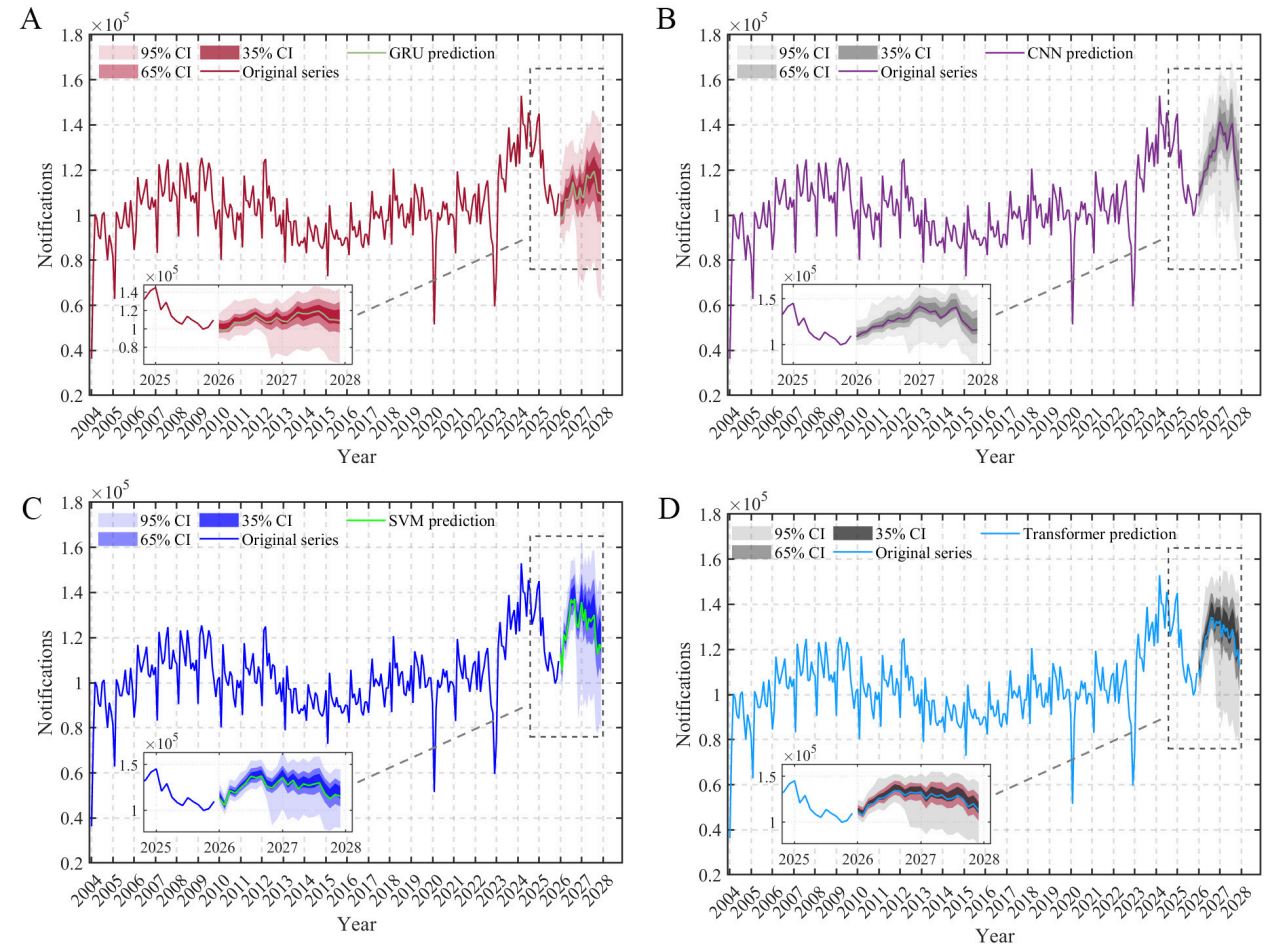
Monthly forecasts of reported hepatitis B cases in mainland China from January 2026 to December 2027. Panels A–D show the prospective month-by-month forecasts generated by the four architectures: GRU, CNN, SVM, and Transformer, respectively. CI – confidence interval, CNN – convolutional neural networks, GRU – gated recurrent units, SVM – support vector machines.

All models consistently project an initial upward trajectory into mid-2026 followed by recurrent seasonal oscillations throughout 2027, they diverge substantially in predicted amplitude and volatility. These discrepancies are most pronounced during the projected August 2026 peak: the SVM and Transformer estimate 135,273 and 134,138 notified cases, respectively, in stark contrast to the markedly more conservative projections of the CNN (n = 125,306) and GRU (n = 114,652). This substantial gap between the most and least conservative models underscores the variations in how each architecture captures peak magnitudes and temporal fluctuations (**Figure 3****;** Tables S6–9 in the **Online Supplementary Document**).

## DISCUSSION

The original time series of reported hepatitis B cases exhibits robust seasonal oscillations. It is characterised by a nadir in February, a dominant peak in March, secondary peaks in July and August, and an overall downward trend throughout late autumn and winter. Gradual changes in the overall disease burden are primarily reflected in the shifting baseline of this consistent seasonal fluctuation. Importantly, the observed long-term trends and periodicities do not solely reflect the virological transmission dynamics of HBV. Unlike respiratory viruses, HBV lacks an obvious temperature driven seasonal dependence. Changes in testing coverage, screening strategies, diagnostic criteria, and reporting procedures all leave discernible signatures in the surveillance data [23]. Therefore, we hypothesise that the seasonal fluctuations primarily arise from calendar driven behavioural patterns. Plausible explanations include holidays and social gatherings that stimulate healthcare seeking or result in high-risk exposures. Other contributing factors involve scheduled periodic medical examinations, workplace screenings, seasonal fluctuations in blood donation and prenatal testing, alongside the operating rhythm of healthcare facilities [24,25]. However, because our study relies on aggregate monthly notifications rather than granular individual level data, these specific behavioural drivers cannot be directly substantiated and must be interpreted strictly as hypotheses. Ultimately, these variations in detection and reporting manifest as cyclical patterns in the number of reported cases.

The monthly anomalies observed during the COVID-19 pandemic, particularly in February 2020 and December 2022, likely represent reporting biases caused by temporary disruptions in healthcare delivery rather than sudden decreases in actual new infections. During this period, public health responses redirected medical resources, scaled down routine outpatient services, shifted care seeking behaviours, and caused widespread diagnostic delays [26,27]. Furthermore, the updated 2022 Hepatitis B guidelines in China proactively lowered the threshold for initiating antiviral treatment to a detectable HBV DNA level above 10 to 20 IU/ml [28]. These operational and anthropogenic changes likely caused cases to be reported in a concentrated or delayed manner instead of reflecting abrupt rises in viral transmission.

Methodologically, decomposing the time series with CEEMDAN effectively isolates a nonlinear and non-stationary series into IMFs and a residual component. This process cleanly separates short-term fluctuations, seasonal oscillations, and long-term trends [29]. Compared with earlier empirical mode decomposition or ensemble empirical mode decomposition variants, CEEMDAN significantly mitigates mode mixing and improves reconstruction accuracy. This enables subsequent predictive models to handle distinct frequency bands separately while minimising the influence of noise [30]. Prior studies confirm that coupling data decomposition with machine learning better captures complex epidemiological signals and improves forecast accuracy [31,32]. Additionally, heuristic population-based optimisers like KOA navigate high-dimensional hyperparameter spaces much more efficiently than empirical tuning or exhaustive grid searches [33]. By balancing exploration and exploitation through a time-adaptive mass gravity attraction mechanism and stochastic updates, KOA avoids suboptimal configurations within a limited evaluation budget [34,35].

A comparative analysis of model goodness-of-fit highlights fundamental differences in information extraction and data recurrence, which justifies the selection of these classic models in this study. The GRU model recurrently propagates hidden states using update and reset gates to selectively retain or discard past information, effectively preventing gradient vanishing or explosion [36,37]. While the GRU model achieved the best fit on the training set in this study, it underperformed on the test set, indicating a certain degree of overfitting. The CNN model applies sliding kernels and pooling layers to extract local temporal patterns or frequency domain features. The local receptive fields are well suited for handling complex data scenarios, but modelling long-range dependencies remains challenging. This limitation may lead to underperformance when simulating over extended periods. The SVM model operates as a kernel-based learner that maps inputs into high dimensional spaces to solve convex optimisation problems. Lacking recurrence or self-attention mechanisms, they are relatively robust against noise and overfitting [38]. However, their limited capacity for long sequence modelling necessitates effective pre-processing, validating the integration of CEEMDAN derived components. Finally, the Transformer model relies on self-attention mechanisms and positional encodings to directly model global long-range dependencies [39]. This ability to flexibly integrate multiscale information is particularly advantageous when processing frequency decomposed inputs, which likely contributed to the superior test set generalisation of the Transformer observed in this study [40]. To further ensure model stability, we implemented rigorous training protocols involving adaptive optimisers, learning rate scheduling, and regularisation techniques such as dropout and weight decay to accelerate convergence and mitigate overfitting [41,42]. Nevertheless, model predictions can heavily depend on the chosen data splitting, forecast horizons, and pre-processing steps. Therefore, rather than declaring a universal methodological hierarchy, we present these empirical rankings as a synthesis of inherent algorithmic capabilities and our specific evaluation configurations. Consequently, goodness-of-fit diagnostics should be interpreted cautiously, and all tuned models retain a certain degree of reference value for forecasting future data.

Admittedly, anomalous months such as February 2020 and December 2022 challenged all architectures and led to systematic underestimations at those specific time points. Nevertheless, the goodness-of-fit diagnostics on the test set confirms that the hybrid CEEMDAN and KOA pipeline performs robustly in terms of predictive accuracy and practical utility. All models achieved the MAPE below 5% and R^2^ above 0.80 [43].

It is crucial to clarify the epidemiological interpretation of the data utilised in this study. According to the criteria of the National Disease Control and Prevention Administration, the monthly reported Hepatitis B cases encompass both acute incident infections and newly diagnosed chronic cases. Given the chronic nature of HBV infection and its prolonged asymptomatic phase, the temporal variations and peaks within this reported series are profoundly influenced by anthropogenic and operational factors. Consequently, the forecasts generated by our models predominantly represent the anticipated administrative reporting volume and the diagnostic burden on the healthcare system. While these models may not track real-time virological transmission, forecasting this reporting volume holds immense practical value. It provides public health authorities with essential foresight for allocating diagnostic resources, managing antiviral drug supply chains, and planning long-term care for newly identified chronic patients.

Looking ahead to the 2026 to 2027 horizon, all models project that the reported burden of Hepatitis B in mainland China is unlikely to decline substantially. The number of reported cases will potentially remain at elevated levels or even rise. Rather than interpreting this as a guaranteed epidemiological resurgence, we frame these projections as a plausible continuation of recent intensified screening and reporting dynamics. These findings underscore the critical need for proactive resource allocation and sustained long-term surveillance by public health authorities.

### Limitations and suggestions

The study also has several limitations that must be acknowledged. First, although the data come from the official reports of the National Disease Control and Prevention Administration, changes in testing, diagnosis, and reporting practices during the COVID-19 pandemic may have affected data completeness and the shape of the time series. Quantifying that precise influence requires further investigation. Second, our focus was solely on the univariate time series. Therefore, we did not incorporate external drivers such as population mobility, environmental factors, or spatial distributions, which may also shape the trends in reported cases. Third, our data partitioning strictly followed the conventional machine learning ratio of 80% for training, 10% for validation, and 10% for testing. However, constrained by the finite total sample size of 264 months, the held-out validation and test sets contained only 25 observations, respectively. This short evaluation horizon provides a limited basis for absolutely stable model comparisons. Finally, the models developed here are designed specifically for short term forecasts. Extending the prediction horizon significantly degrades performance, and sudden unexpected events that lack historical analogues are inherently difficult for these algorithms to anticipate.

Based on our findings, we offer three recommendations for future disease prediction and public health decision making. First, model outputs should always be interpreted in tandem with the operational context of the surveillance system. Any artificial fluctuations stemming from changes in testing availability or reporting rules should be identified and, where necessary, corrected using compensatory screening or retrospective data audits. Second, surveillance systems should be made more resilient. For example, authorities could maintain a minimum screening and reporting capacity for key infectious diseases during public health emergencies. Establishing rapid supplementary reporting and calibration mechanisms would also reduce the impact of such disruptive events on long term trend assessments. Third, predictive modelling efforts should proactively support resource allocation and targeted interventions. These forecasts can directly inform the optimal deployment of vaccines, prenatal screening, and follow up care for high-risk populations. Ultimately, quantitative forecasts must be combined with field epidemiological findings to effectively prioritise public health interventions.

## CONCLUSIONS

This nationwide study demonstrates that coupling CEEMDAN data decomposition with KOA optimised architectures yields reliable short to medium term forecasts of reported Hepatitis B cases in mainland China. The Transformer consistently delivered the most accurate out of sample predictions, while the GRU, CNN, and SVM captured complementary temporal scales. Forecasts for 2026 to 2027 indicate that reported cases will likely remain elevated, underscoring the critical need for sustained surveillance and proactive resource planning to manage the enduring clinical burden. By integrating data decomposition, heuristic optimisation, and advanced modelling, this framework strengthens the continuous loop of surveillance, prediction, and decision making, offering a practical tool to guide public health interventions.

## Additional material


Online Supplementary Document

## Data Availability

**Data availability:** All raw data analysed during this study are included in the supplementary materials. The original source data are publicly accessible and were retrieved from the official Monthly Overview of Notifiable Infectious Disease published by the National Disease Control and Prevention Administration. For any further inquiries or additional data requests, please contact the corresponding author.
