# Supplementary material for: CEEMDAN-decomposed time series forecasting of reported hepatitis B cases using KOA-optimised deep learning: a nationwide study in mainland China (2004–2027)
Source: J Glob Health. 2026 Sep 18;16:04200. doi: 10.7189/jogh.16.04200 (PMC13587761; doi:10.7189/jogh.16.04200)
Supplement: Supplementary file 1 [file jogh-16-04200-s001.pdf]

Supplement to: Wang Z, Bi F, Duan Z, Ding L, Song B, Wang K, Zhao J, Li H. CEEMDAN-decomposed time series forecasting of reported hepatitis B cases using KOA-optimised deep learning: a nationwide study in mainland China (2004–2027). J Glob Health. 2026;16:04200.

Original time series of reported Hepatitis B cases

Supplementary Table 1 illustrates the original time-series data collected in this study, together with the seasonal fluctuation components and long-term trend derived from the analysis.

Supplementary Table 1. Original time series and the seasonal decomposition and long-term trend

Time	Raw data	Seasonality	Long-term trend	Time	Raw data	Seasonality	Long-term trend
2004-01	36301	-5178.504	88528.750	2015-01	96649	-5178.504	90395.833
2004-02	75937	-11476.712	88528.750	2015-02	72869	-11476.712	90107.958
2004-03	100375	13095.530	88528.750	2015-03	104427	13095.530	89988.458
2004-04	98138	4386.712	88528.750	2015-04	94350	4386.712	90087.458
2004-05	90822	2131.409	88528.750	2015-05	91194	2131.409	90193.958
2004-06	89361	-1226.082	88528.750	2015-06	89224	-1226.082	90355.000
2004-07	100026	6496.034	88528.750	2015-07	93586	6496.034	90136.500
2004-08	101221	6343.646	89872.458	2015-08	89228	6343.646	90235.875
2004-09	89089	-3141.665	89370.667	2015-09	89806	-3141.665	90679.750
2004-10	80007	-6015.902	89442.333	2015-10	86393	-6015.902	90686.333
2004-11	91169	-1731.120	89613.833	2015-11	87284	-1731.120	90799.875
2004-12	87203	-3683.345	89958.458	2015-12	90103	-3683.345	91001.000
2005-01	81693	-5178.504	90097.500	2016-01	89699	-5178.504	90941.625
2005-02	62794	-11476.712	90226.375	2016-02	82204	-11476.712	91194.750
2005-03	101475	13095.530	90703.292	2016-03	105745	13095.530	91445.833
2005-04	98758	4386.712	91594.833	2016-04	93190	4386.712	91307.125
2005-05	94318	2131.409	92841.125	2016-05	95079	2131.409	91443.833
2005-06	94136	-1226.082	93942.208	2016-06	90166	-1226.082	91671.417
2005-07	98588	6496.034	94516.917	2016-07	91219	6496.034	91597.500
2005-08	105752	6343.646	95886.833	2016-08	97670	6343.646	92187.958
2005-09	96004	-3141.665	97780.083	2016-09	87390	-3141.665	93112.333
2005-10	94489	-6015.902	98860.417	2016-10	85480	-6015.902	93525.042
2005-11	106598	-1731.120	99728.292	2016-11	91478	-1731.120	94009.917
2005-12	98200	-3683.345	100746.333	2016-12	91371	-3683.345	94705.458
2006-01	84489	-5178.504	101891.875	2017-01	86657	-5178.504	95425.083
2006-02	92876	-11476.712	102872.083	2017-02	99417	-11476.712	95991.292
2006-03	116831	13095.530	103616.625	2017-03	110717	13095.530	96648.500
2006-04	109330	4386.712	104265.417	2017-04	98123	4386.712	97193.958
2006-05	104575	2131.409	104654.333	2017-05	101783	2131.409	97604.000
2006-06	108312	-1226.082	104929.125	2017-06	100155	-1226.082	98120.875
2006-07	111905	6496.034	106080.708	2017-07	98501	6496.034	99310.583

2006-08	115960	6343.646	106614.708	2017-08	103977	6343.646	99720.292
2006-09	103665	-3141.665	106451.542	2017-09	96856	-3141.665	99612.417
2006-10	102399	-6015.902	106925.042	2017-10	89105	-6015.902	100371.458
2006-11	108022	-1731.120	107312.250	2017-11	97694	-1731.120	101009.917
2006-12	103371	-3683.345	107540.500	2017-12	97560	-3683.345	101234.250
2007-01	106956	-5178.504	107938.333	2018-01	109021	-5178.504	101386.083
2007-02	83225	-11476.712	108623.667	2018-02	86886	-11476.712	101652.708
2007-03	122566	13095.530	109092.625	2018-03	120659	13095.530	101633.833
2007-04	114959	4386.712	109363.708	2018-04	106398	4386.712	101782.500
2007-05	108239	2131.409	109803.250	2018-05	108831	2131.409	102126.958
2007-06	110126	-1226.082	110339.833	2018-06	98491	-1226.082	102207.417
2007-07	119639	6496.034	110656.208	2018-07	103809	6496.034	102103.625
2007-08	124674	6343.646	111012.250	2018-08	105068	6343.646	102221.625
2007-09	106206	-3141.665	111340.583	2018-09	95312	-3141.665	102112.500
2007-10	106364	-6015.902	111526.583	2018-10	94217	-6015.902	101993.750
2007-11	114606	-1731.120	111892.875	2018-11	100849	-1731.120	102054.917
2007-12	109665	-3683.345	111982.083	2018-12	96336	-3683.345	101907.875
2008-01	108255	-5178.504	112019.292	2019-01	107754	-5178.504	102221.042
2008-02	90471	-11476.712	111803.708	2019-02	90985	-11476.712	102661.125
2008-03	123200	13095.530	111425.917	2019-03	113941	13095.530	102845.292
2008-04	118789	4386.712	111521.583	2019-04	110266	4386.712	103139.458
2008-05	113200	2131.409	111235.542	2019-05	106431	2131.409	103384.542
2008-06	107306	-1226.082	110867.083	2019-06	97362	-1226.082	103682.042
2008-07	123352	6496.034	110150.917	2019-07	112454	6496.034	103227.333
2008-08	115787	6343.646	110684.958	2019-08	106985	6343.646	100885.375
2008-09	106026	-3141.665	112048.708	2019-09	97815	-3141.665	98165.792
2008-10	108840	-6015.902	112221.167	2019-10	98774	-6015.902	96716.000
2008-11	105265	-1731.120	112193.208	2019-11	102174	-1731.120	95975.000
2008-12	110163	-3683.345	112466.125	2019-12	102151	-3683.345	95690.708
2009-01	90569	-5178.504	112842.625	2020-01	91026	-5178.504	95508.958
2009-02	120974	-11476.712	113012.667	2020-02	51506	-11476.712	95050.625
2009-03	125427	13095.530	113234.042	2020-03	88150	13095.530	95170.667
2009-04	120701	4386.712	112934.000	2020-04	101262	4386.712	95354.875
2009-05	110617	2131.409	112101.750	2020-05	97651	2131.409	95156.792
2009-06	116439	-1226.082	111239.750	2020-06	99319	-1226.082	95008.667
2009-07	123255	6496.034	111358.542	2020-07	106135	6496.034	95229.042
2009-08	119965	6343.646	110153.125	2020-08	102304	6343.646	96843.167
2009-09	107161	-3141.665	108102.708	2020-09	105377	-3141.665	99459.042
2009-10	100504	-6015.902	107086.500	2020-10	95633	-6015.902	101142.208
2009-11	93627	-1731.120	106004.250	2020-11	100561	-1731.120	101844.917
2009-12	101113	-3683.345	104849.583	2020-12	100209	-3683.345	102263.917
2010-01	102470	-5178.504	103349.750	2021-01	98257	-5178.504	102616.667
2010-02	80143	-11476.712	101883.792	2021-02	83014	-11476.712	102862.875
2010-03	117048	13095.530	100614.917	2021-03	119423	13095.530	102699.583

2010-04	104691	4386.712	99575.083	2021-04	110385	4386.712	102559.292
2010-05	100653	2131.409	99415.458	2021-05	105393	2131.409	102657.042
2010-06	98691	-1226.082	99585.625	2021-06	101633	-1226.082	103073.083
2010-07	105007	6496.034	99374.750	2021-07	112287	6496.034	103874.000
2010-08	103030	6343.646	99540.083	2021-08	102061	6343.646	104917.625
2010-09	93643	-3141.665	99790.542	2021-09	101701	-3141.665	105265.667
2010-10	89066	-6015.902	99885.458	2021-10	95942	-6015.902	104437.667
2010-11	101234	-1731.120	100193.083	2021-11	102598	-1731.120	103732.125
2010-12	97590	-3683.345	100615.500	2021-12	108157	-3683.345	103845.958
2011-01	100932	-5178.504	100939.667	2022-01	109531	-5178.504	104078.208
2011-02	85649	-11476.712	101494.458	2022-02	96787	-11476.712	104648.000
2011-03	117553	13095.530	102200.167	2022-03	114003	13095.530	105177.000
2011-04	106464	4386.712	102925.125	2022-04	95933	4386.712	104904.292
2011-05	106263	2131.409	103612.583	2022-05	102912	2131.409	103981.208
2011-06	103219	-1226.082	104107.250	2022-06	106846	-1226.082	101277.625
2011-07	108259	6496.034	103881.417	2022-07	112648	6496.034	97802.625
2011-08	113093	6343.646	104976.083	2022-08	115375	6343.646	97158.250
2011-09	100517	-3141.665	106848.417	2022-09	101083	-3141.665	98500.125
2011-10	99591	-6015.902	107194.125	2022-10	90015	-6015.902	99891.625
2011-11	107208	-1731.120	107520.583	2022-11	86371	-1731.120	101287.000
2011-12	103488	-3683.345	107739.625	2022-12	59498	-3683.345	101963.625
2012-01	89614	-5178.504	107664.333	2023-01	74790	-5178.504	102617.167
2012-02	123239	-11476.712	107408.750	2023-02	116063	-11476.712	104115.833
2012-03	124899	13095.530	106925.292	2023-03	126932	13095.530	106028.833
2012-04	107415	4386.712	106526.875	2023-04	116400	4386.712	108588.750
2012-05	113147	2131.409	106072.208	2023-05	115934	2131.409	112127.292
2012-06	101592	-1226.082	105278.625	2023-06	110063	-1226.082	116619.625
2012-07	108079	6496.034	105308.042	2023-07	125116	6496.034	121744.625
2012-08	107139	6343.646	103991.292	2023-08	138875	6343.646	124569.625
2012-09	94868	-3141.665	101419.667	2023-09	123495	-3141.665	125934.292
2012-10	95678	-6015.902	100271.583	2023-10	129041	-6015.902	128025.917
2012-11	100209	-1731.120	99173.292	2023-11	132270	-1731.120	130015.625
2012-12	91441	-3683.345	97863.875	2023-12	121415	-3683.345	131796.750
2013-01	102367	-5178.504	96783.250	2024-01	135873	-5178.504	133445.583
2013-02	78884	-11476.712	95933.792	2024-02	122780	-11476.712	134344.917
2013-03	107535	13095.530	95239.542	2024-03	152967	13095.530	134488.250
2013-04	97225	4386.712	94553.292	2024-04	140564	4386.712	134569.917
2013-05	96978	2131.409	93668.000	2024-05	139523	2131.409	134595.167
2013-06	86335	-1226.082	93019.583	2024-06	129221	-1226.082	135471.542
2013-07	97401	6496.034	92353.375	2024-07	145530	6496.034	136693.000
2013-08	97430	6343.646	92021.167	2024-08	140045	6343.646	136991.917
2013-09	87915	-3141.665	91852.042	2024-09	125765	-3141.665	135908.625
2013-10	86161	-6015.902	91406.208	2024-10	128731	-6015.902	133801.750
2013-11	88479	-1731.120	91093.750	2024-11	133186	-1731.120	131390.583

2013-12	87609	-3683.345	90961.417	2024-12	141532	-3683.345	129079.500
2014-01	90210	-5178.504	90966.125	2025-01	145071	-5178.504	126740.333
2014-02	83068	-11476.712	90753.292	2025-02	120756	-11476.712	124130.250
2014-03	99292	13095.530	90609.833	2025-03	128992	13095.530	122025.083
2014-04	94768	4386.712	90599.292	2025-04	113974	4386.712	119986.750
2014-05	91936	2131.409	90452.667	2025-05	108245	2131.409	117463.542
2014-06	88201	-1226.082	90345.750	2025-06	105033	-1226.082	114820.917
2014-07	95648	6496.034	90646.875	2025-07	113578	6496.034	114820.917
2014-08	94075	6343.646	90490.208	2025-08	109355	6343.646	114820.917
2014-09	87827	-3141.665	90279.208	2025-09	105931	-3141.665	114820.917
2014-10	85996	-6015.902	90475.750	2025-10	99645	-6015.902	114820.917
2014-11	85125	-1731.120	90427.417	2025-11	101715	-1731.120	114820.917
2014-12	88397	-3683.345	90439.125	2025-12	109580	-3683.345	114820.917

The architectures of deep learning models

The GRU model adopted the depth and flow of a stacked LSTM architecture but substituted GRU cells for the LSTM units, arranging two recurrent layers with a dropout layer after each to capture temporal dependencies while controlling overfitting; their output was aggregated by a fully connected layer before the regression head generated the next-month forecast. The CNN was built as a temporal convolutional network tailored to the univariate sliding-window setup: an initial convolutional layer applied multiple filters along the time dimension to capture local motifs, followed by batch normalization and max-pooling to stabilize training and reduce dimensionality; this normalization–pooling sequence was repeated, a dropout layer was inserted for regularization, and the resulting feature maps were flattened, passed through a fully connected layer, and finally fed to a regression output layer for the prediction. The Transformer used only the encoder to model long-range dependencies over the 12-month window: raw inputs were embedded into dense vectors, positional encodings were added to retain order, and the combined embeddings went through a multi-head self-attention layer with dropout regularization; this attention/dropout block was repeated once to deepen temporal extraction, then a fully connected layer assimilated the processed features before the regression output layer produced the next-month forecast (**Supplementary Figure 1**).

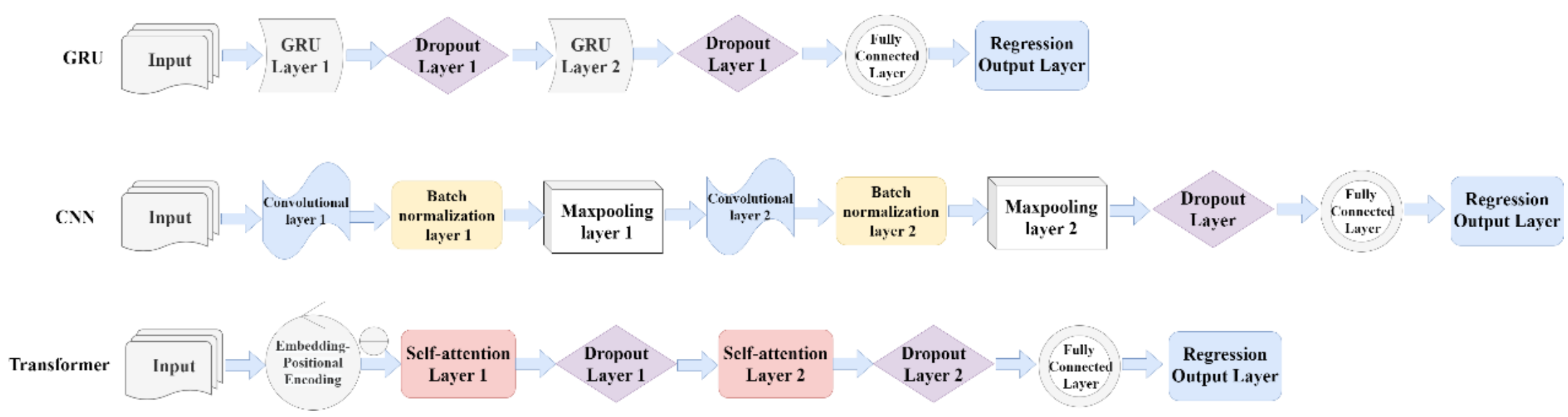

Supplementary Figure 1. The deep learning model structures used in this study.

Hyperparameter search based on the KOA

At each iteration, KOA computes the Euclidean distance between every agent and the Sun and converts fitness values into relative "masses" so that better agents exert stronger attraction; a global mass

coefficient decays exponentially with iteration count ($M = M0 \cdot \exp(-\lambda \cdot t/Tmax)$) to favour exploration early and exploitation later. Distances and masses are normalised and used to compute a mass-based attraction term; candidate updates then follow one of two stochastic branches—a recombination branch, which forms an adaptive average from the current agent, a randomly selected peer and the sun and applies a masked replacement to favour intensification, and a velocity-like branch, which constructs a velocity scale dependent on the decaying mass, the combined masses and an orbital geometry term and combines velocity, random perturbations and gravitational attraction to produce exploratory or exploitative moves according to proximity to the Sun. Updated positions that fall outside the feasible domain are projected back into bounds by clipping or random reinitialisation. After each proposed move, the new candidate is evaluated and an elitist acceptance rule is applied: a change is accepted only if it improves the agent's objective, and any accepted improvement that is globally superior updates the Sun; the best-so-far score is recorded after every function evaluation to produce a convergence trace.

Hyperparameter search was conducted with a population size of three and three evolution iterations, optimizing for the lowest validation RMSE. The parameter search ranges were as follows: for the GRU model, the number of hidden units per layer was searched within 2 to 50, while dropout ranged from 0.001 to 0.5; for the CNN model, the number of neurons per convolutional layer varied between 2 and 50; for the SVM model, the kernel scale spanned 0.01 to 5, the box constraint 0.01 to 30, and epsilon 0.001 to 1; for the Transformer model, the positional-encoding dimension was explored between 0 and 6, both attention heads and key dimension ranged from 1 to 64, and dropout varied from 0.001 to 0.5.

The hyperparameter optimization process was executed using a strictly controlled computational budget to ensure a fair evaluation. The algorithm was configured with a population size of three and exactly three evolution iterations, specifically optimizing to minimize the validation RMSE. To standardize the training dynamics, the learning rate for all deep learning architectures (the GRU, CNN, and Transformer) was kept strictly constant at 0.001. The specific parameter search spaces were then tailored to the distinct architecture of each model. For the GRU model, the number of hidden neurons per layer was searched within a range of 2 to 50, and the dropout rate was tuned between 0.001 and 0.5. For the CNN model, the number of filters per convolutional layer varied from 2 to 50, and the dropout rate was evaluated between 0.001 and 0.5, with the convolutional stride kept constant at 1. Regarding the SVM model, the search space spanned from 0.01 to 5 for the kernel scale, 0.01 to 30 for the box constraint, and 0.001 to 1 for the epsilon parameter. Finally, for the Transformer architecture, the positional encoding dimension was explored between 0 and 6. Additionally, both the number of attention heads and the key dimension ranged from 1 to 64, and the dropout rate was tuned from 0.001 to 0.5 (**Supplementary Table 2**).

Supplementary Table 2. Optimized hyperparameters and search ranges for each model based on the KOA

Models/ Algorithm	Parameters	Search Space / Settings	Optimal Values
**KOA Settings**	Maximum iterations	500	-
	Population size	3	-
	Number of evolutions	3	-
	Termination criteria	1. Maximum iterations reached	-

		2. Fitness convergence (no significant change or stable positions)	
**GRU**	Number of hidden neurons per layer	2 - 50	35, 35
	Learning rate	0.001	-
	Dropout rate	0.001 - 0.5	0.031
**CNN**	Number of filters per convolutional layer	2 - 50	6, 47
	Learning rate	0.001	-
	Dropout rate	0.001 - 0.5	0.02
	Stride	1	-
**SVM**	Kernel scale (γ)	0.01 - 5	0.01
	Box constraint (C)	0.01 - 30	0.01
	Epsilon (ε)	0.001 - 1	0.001
**Transformer**	Positional encoding dimension	0 - 6	1
	Number of attention heads and keys	1 - 64	2
	Dropout rate	0.001 - 0.5	0.001
	Learning rate	0.001	-

Parameter settings and data decomposition results based on CEEMDAN

The input series was first standardized by its sample standard deviation. We set the noise amplitude parameter to 0.2, so that at each step the added Gaussian noise had a standard deviation equal to 20% of the standard deviation of the current signal. We used 100 independent noise realizations: for each intrinsic mode function, EMD was applied separately to 100 noise-contaminated versions of the signal, and the resulting modes were averaged to obtain a single IMF. CEEMDAN iterations continued while the current residual contained more than two extrema, indicating the presence of an oscillatory component; once the residual became nearly monotonic, it was taken as the final mode. Within each CEEMDAN step, EMD used the standard Huang–Rilling stopping criterion with parameters threshold and tolerance for 0.05, enforcing local symmetry of the upper and lower envelopes and a near-match between the numbers of extrema and zero crossings before accepting a mode as an IMF. The maximum number of sifting iterations per IMF was limited to 10; if the stopping criterion was not satisfied earlier, sifting was forcibly terminated after 10 iterations and the current component was retained.

The CEEMDAN decomposition of the monthly hepatitis B series separated the original signal into seven intrinsic mode functions (IMF1–IMF7) and a residual, with components ordered from highest to lowest characteristic frequency. IMF1 captures high-frequency, small-amplitude month-to-month variability and transient perturbations; IMF2 and IMF3 represent intermediate-frequency oscillations that reflect sub-annual and intra-annual fluctuations. IMF4 exhibits a clear quasi-periodic waveform consistent with an annual seasonal cycle, while IMF5 corresponds to lower-frequency, multi-year undulations. IMF6 and IMF7 are the slowest components and together with the residual define the long-term baseline and multi-year trend of the series, with IMF7 in particular showing a gradual upward drift. Notably, brief disturbances such as the short-term anomalies around 2020 are resolved primarily in the high-frequency

IMFs, whereas sustained changes in baseline incidence appear in the lowest-frequency components. By disentangling noise, seasonality and long-term trend across distinct time scales, CEEMDAN provides a principled basis for modelling each scale separately and thereby improving the interpretability and predictive performance of subsequent CEEMDAN-KOA forecasts (**Supplementary Table 3, Supplementary Figure 2**).

Supplementary Table 3. Data decomposition based on the CEEMDAN method

Time	IMF1	IMF2	IMF3	IMF4	IMF5	IMF6	IMF7
2004-01	-20255.33661	-6591.549235	-14047.47877	-3645.254758	-500.1939042	-8818.055626	244.8515785
2004-02	6400.962346	-1947.213024	-7667.661356	-2096.109489	-272.1368168	-8869.573587	423.2008951
2004-03	17692.72211	1085.31236	403.0625591	-504.9164595	-57.28407736	-8879.270659	616.393503
2004-04	5247.682699	1880.348388	7650.353896	1225.832754	75.97181948	-8840.386449	823.8735224
2004-05	-7165.935283	2116.280924	10324.96821	2997.66446	114.7430624	-8742.056044	1044.924326
2004-06	-10390.33084	1847.641866	11112.11492	3826.568998	68.61479062	-8572.528198	1278.871197
2004-07	2483.264346	1077.498383	9724.750124	3313.525635	-25.50885476	-8322.755064	1525.194451
2004-08	6556.674778	570.4464582	7823.474616	2277.966391	-114.3887192	-7987.818975	1783.497415
2004-09	-2663.03347	744.2589765	5394.326522	935.4596159	-181.8890752	-7566.858644	2053.57303
2004-10	-7149.629327	1104.923146	1221.377721	-611.1886982	-266.9582666	-7062.573734	2335.204047
2004-11	7955.091196	1107.109582	-2052.759619	-2094.691321	-391.163917	-6481.566923	2627.977442
2004-12	9144.288398	570.191488	-6416.521448	-3237.509291	-515.3452737	-5836.081223	2931.473838
2005-01	7179.658	-1715.333112	-8513.900751	-3392.161211	-594.5230085	-5142.24337	3245.239971
2005-02	-14475.33077	-2505.175632	-7035.344314	-2428.734355	-605.8707804	-4414.715543	3568.949386
2005-03	13872.02175	563.5474887	-2504.072494	-885.1236982	-562.5169	-3665.395054	3902.217928
2005-04	2293.127869	1852.06884	2079.721163	879.3799583	-503.565725	-2905.638423	4244.407425
2005-05	-5227.989872	483.8336055	3774.827759	2411.569161	-454.2123995	-2147.330867	4594.610769
2005-06	-6302.677956	-557.045068	3524.926064	3386.375908	-412.8086182	-1401.410039	4951.80973
2005-07	-1602.095043	-300.498901	1608.418864	3575.414996	-339.7375316	-679.3465802	5315.00656
2005-08	5381.760169	-92.07834085	1175.002761	2710.602261	-190.7248027	9.539484234	5683.264887
2005-09	-4423.863994	28.35886315	1515.841267	987.1935665	44.54046446	658.003508	6055.785856
2005-10	-5390.915695	197.5304585	1367.605597	-905.7952465	327.7463915	1259.934301	6431.608652
2005-11	8945.139663	393.7924649	-618.5345366	-2646.278897	638.0058507	1812.063303	6809.80915
2005-12	3418.000333	-65.49482695	-2848.360881	-4067.933188	932.0523595	2316.041538	7189.455705
2006-01	-11143.27177	-777.9087543	-2201.88471	-4264.398598	1143.690813	2775.295037	7569.534375
2006-02	-5946.83152	-163.7778828	-2107.814923	-2718.326381	1220.166191	3194.605917	7948.904248
2006-03	11194.74611	452.0729271	693.1684666	-37.04396271	1113.653651	3578.464058	8326.335531
2006-04	-830.722859	327.1748756	2261.083665	2517.151755	854.0791833	3931.078287	8700.646701
2006-05	-7712.823708	-251.4226565	2771.010928	4258.941312	554.517096	4255.198017	9070.823497
2006-06	-4945.255432	-439.7999832	2816.649374	4894.05497	311.7380214	4551.378607	9435.930381
2006-07	-1643.896685	295.9390188	2401.453943	4366.618684	128.266116	4816.425698	9795.080521
2006-08	5201.86946	730.6407366	224.6954627	2837.107375	-33.62637887	5049.752004	10147.42333
2006-09	-3157.906705	-565.5197752	-535.7535465	522.1465907	-203.0633168	5254.850527	10491.91678
2006-10	-2024.324874	-1240.95809	42.35267674	-2165.041077	-385.4753336	5431.32164	10827.48838
2006-11	3573.897554	658.2010242	-63.46566795	-4295.861866	-546.7920926	5574.840478	11153.16975
2006-12	181.3851189	2317.257591	-2575.26713	-5103.864237	-620.3447636	5682.310068	11468.11904

2007-01	9429.527724	-1168.817856	-5736.370674	-4610.701671	-557.3195879	5754.326061	11771.58612
2007-02	-13716.69112	-3171.876167	-6088.393868	-3421.208824	-359.9615887	5794.995761	12063.07423
2007-03	15941.88618	-112.3459672	-2056.486282	-1471.174355	-63.46313474	5809.986821	12342.35386
2007-04	-1065.602104	1671.627501	2213.919989	1245.620413	250.9518502	5808.90873	12609.28643
2007-05	-14026.6713	1293.027629	6009.846763	3574.461084	453.8061644	5798.728066	12863.61488
2007-06	-12186.20956	384.63903	5729.832266	4519.147359	474.1073771	5780.350501	13105.17151
2007-07	61.17340962	-261.9234261	3753.075986	4293.252558	342.7657323	5752.158466	13333.85032
2007-08	9177.914086	-449.7948443	823.3604858	3344.467067	107.4694426	5711.929505	13549.38333
2007-09	-5483.031622	-557.1954017	-1208.561259	1728.428603	-137.76882	5659.517303	13751.81113
2007-10	-3788.017306	-528.3283092	-699.0591094	-326.0108771	-322.6099508	5591.486845	13941.35019
2007-11	5919.621698	807.0345141	-1589.809487	-2246.624277	-438.8878855	5500.073308	14118.21718
2007-12	3302.966126	1441.724948	-3230.83423	-3555.814204	-529.8286007	5378.089304	14282.40244
2008-01	6335.844138	-1039.840529	-4795.420037	-3854.200643	-662.1344648	5222.030861	14433.83817
2008-02	-12260.63357	-2031.953491	-3547.455855	-3091.219508	-852.5251552	5030.298747	14572.40712
2008-03	13371.76295	563.4494841	-519.2465955	-1333.974943	-1071.111323	4803.277018	14698.00995
2008-04	3289.763974	1147.497509	2787.618504	744.8744721	-1257.151401	4543.628857	14810.68068
2008-05	-3804.598057	-73.50423134	3997.103785	2488.418969	-1330.928882	4258.09567	14910.60574
2008-06	-10037.34462	-298.6442512	3861.800179	3270.157032	-1230.599944	3956.576028	14998.09552
2008-07	7110.494984	263.5149461	2485.786166	2909.350307	-954.0352717	3647.906853	15073.46809
2008-08	2217.038046	-88.3678173	1110.194459	1761.185454	-529.3290793	3335.842108	15137.00695
2008-09	-3614.292963	-699.1382181	-927.5236069	210.1044894	-23.47830135	3021.695278	15188.96382
2008-10	767.0877561	-26.78484553	-1650.24273	-1578.973073	498.1161097	2707.033055	15229.56462
2008-11	-1086.014231	1358.215959	-3471.574918	-3098.423862	992.7683165	2394.180103	15258.86416
2008-12	7874.717589	-337.783576	-5616.741454	-3513.984181	1456.92291	2084.999241	15276.88074
2009-01	-10984.6053	-1566.258851	-6209.409462	-2596.353477	1905.68554	1778.950428	15283.81093
2009-02	12570.57161	74.86560752	-2824.317686	-940.3438765	2365.423339	1473.38604	15279.86832
2009-03	9715.306675	832.6433676	1766.068803	838.3052951	2856.884103	1162.506283	15265.15393
2009-04	513.1020608	266.2661736	5081.790366	2432.088119	3321.306457	842.7191701	15239.74667
2009-05	-11012.39905	-72.42538091	6047.651481	3293.015313	3631.646287	509.6222489	15203.72857
2009-06	-5072.773736	-42.02470276	6418.986269	3132.13496	3661.081037	157.3800842	15157.47319
2009-07	3526.333714	121.323415	6079.16347	2270.300135	3338.076306	-217.445697	15101.45555
2009-08	4563.282571	92.04375946	3901.004368	1266.990699	2677.485318	-614.9745684	15035.80822
2009-09	-2713.911066	-268.8845517	1123.516282	254.3749002	1784.700632	-1028.689332	14960.41669
2009-10	-2458.441189	-51.97000685	-3257.260081	-990.9536553	780.7991921	-1447.279963	14874.93543
2009-11	-4811.373069	807.3166386	-5720.536566	-2365.738331	-261.8582601	-1857.318954	14779.03808
2009-12	7038.702032	175.1541349	-7305.706912	-3003.595194	-1277.70768	-2245.741664	14672.48833
2010-01	11276.49026	-1124.064065	-8055.336181	-2433.511242	-2205.501297	-2603.183497	14555.09189
2010-02	-13954.25723	-1272.236546	-5329.771012	-867.828269	-2999.46852	-2919.496871	14426.71984
2010-03	14121.09893	844.8885412	1037.953587	511.5845015	-3627.882748	-3184.263959	14287.18723
2010-04	-3879.06627	923.9162415	6559.715778	1343.153256	-4058.76299	-3388.495219	14136.17631
2010-05	-8132.905688	-183.4321434	7548.337427	2193.471046	-4269.487446	-3526.324468	13973.14061
2010-06	-8231.38783	-135.6198117	5484.864716	2603.994346	-4279.829907	-3593.684895	13797.65832
2010-07	2017.770021	176.3323969	2004.125801	1886.350534	-4139.130586	-3587.085546	13609.83744
2010-08	5041.527716	-53.27698051	-1484.301746	503.0552068	-3914.701753	-3503.914142	13410.01819

2010-09	-151.0045001	-664.971996	-3842.15451	-924.3951273	-3655.243481	-3341.085298	13198.49021
2010-10	-4551.650845	-825.0465826	-2959.017537	-2110.454128	-3376.804254	-3100.592673	12975.47141
2010-11	4996.806042	1406.079965	-2010.429356	-3053.692841	-3057.870835	-2791.725018	12741.04932
2010-12	405.8713841	2047.087024	-2117.587148	-3130.609353	-2674.892403	-2427.74127	12495.41656
2011-01	6806.711887	-2275.86972	-2589.738919	-1977.130454	-2231.885771	-2019.010106	12238.78149
2011-02	-10740.12408	-2973.223982	-2083.99151	-159.6214068	-1756.645185	-1575.422004	11971.15691
2011-03	10873.9096	2057.106754	667.2409237	1718.689265	-1298.04034	-1111.170242	11692.58263
2011-04	-5298.598374	2281.083896	3525.92059	3123.668679	-864.3124037	-644.7268715	11403.3515
2011-05	-2569.324229	-835.0170938	2314.424273	3930.179382	-408.5780543	-194.4119349	11104.01499
2011-06	-4451.610451	-1306.388627	1051.837735	3875.837188	126.6350275	222.230182	10795.44718
2011-07	-66.45897076	-82.01860246	1224.950312	2461.774029	759.3912608	595.1457324	10478.6721
2011-08	4486.876093	734.5275788	2553.374165	-102.9448724	1472.26197	924.7702528	10154.80207
2011-09	-5878.678856	1047.509561	1860.144094	-2622.271358	2217.270981	1217.425181	9825.214082
2011-10	-2213.17243	332.8670108	-945.004318	-4284.095272	2896.321327	1481.934875	9491.443447
2011-11	10118.91556	-1208.068731	-4100.915822	-4719.252671	3429.628448	1722.301434	9155.103136
2011-12	6850.240553	-2161.81021	-4961.028173	-3553.549693	3771.904236	1935.287781	8817.821572
2012-01	-12541.47834	-1591.91783	-2271.238318	-1267.213743	3922.018204	2115.697579	8480.891827
2012-02	11050.12588	1501.310339	2349.943014	1272.648704	3916.239246	2258.817204	8145.291575
2012-03	7884.771007	2815.324003	4245.900507	3232.957519	3826.423429	2360.68566	7811.645252
2012-04	-7718.477657	752.6148525	3951.013432	4131.781527	3702.715951	2417.957167	7480.138146
2012-05	5633.400962	-2195.967729	-303.275693	4201.804394	3557.311344	2430.360116	7150.799211
2012-06	-4558.288354	-1268.426929	-1165.315934	3343.54496	3374.208576	2395.494081	6823.47756
2012-07	2085.484843	1094.902483	-1157.859938	1555.980524	3069.152078	2311.896458	6497.863025
2012-08	3415.663349	1070.662294	-567.0085599	-270.9271829	2540.896406	2180.602841	6173.616635
2012-09	-3708.526226	-795.3056842	-1102.713179	-1709.158753	1756.819907	2007.389384	5850.354328
2012-10	-109.9927814	-1673.676498	-279.1697563	-2917.54675	792.7442784	1795.410319	5527.622257
2012-11	4088.057845	214.8742761	208.2065348	-3378.450375	-194.9540404	1550.329875	5204.931785
2012-12	-6311.753106	2715.932634	262.1132028	-2816.750025	-1058.429732	1278.614508	4881.767067
2013-01	9428.873207	-577.8543103	-924.6640824	-1870.030924	-1694.911376	984.6056807	4557.660436
2013-02	-11388.84976	-3431.372804	-735.4019025	-820.1179624	-2085.131897	674.9710976	4232.244636
2013-03	11684.32755	1204.452899	-228.1345835	488.0242344	-2290.744901	358.9488383	3905.416601
2013-04	-2359.152526	2934.409601	1157.553856	1864.56099	-2383.979221	45.71153792	3577.246942
2013-05	747.5950573	-443.961884	877.9804263	2852.946627	-2410.935585	-259.5968562	3248.328549
2013-06	-7529.825446	-1246.40679	-89.43460258	2881.374005	-2394.267354	-550.014525	2919.7358
2013-07	3677.498887	267.8497846	-162.1529731	1832.186462	-2309.090918	-821.7667948	2593.122642
2013-08	4102.178029	567.2102503	1185.642716	207.5058842	-2132.777494	-1074.604968	2270.581895
2013-09	-2228.187935	3.430561229	191.959866	-1123.545749	-1863.183932	-1306.5118	1954.463976
2013-10	-1897.071856	-426.0434835	-411.236593	-1988.230854	-1522.669218	-1511.104557	1647.084818
2013-11	1038.480041	45.73164867	-902.170301	-2453.621208	-1173.020636	-1682.329497	1350.584759
2013-12	422.8999879	422.2430082	-1249.870864	-2621.910763	-857.363459	-1815.617358	1066.824393
2014-01	3216.665473	-463.2462323	-799.6972689	-2286.349676	-575.1940578	-1909.147388	797.3438616
2014-02	-5597.556825	-432.8866585	-236.6070412	-1134.690729	-329.581496	-1962.906472	543.392433
2014-03	6118.561885	765.3136388	1372.746502	615.6352306	-119.3141414	-1976.466756	306.0987394
2014-04	446.8689331	616.3426306	990.0841225	2296.512699	79.76073	-1949.547911	86.60925825

2014-05	-1865.178117	-548.1049619	608.5828093	3258.19883	284.0147411	-1882.037289	-114.1203616
2014-06	-4651.623878	-518.9661979	-454.2595268	3206.235216	499.9160914	-1773.986268	-295.5316181
2014-07	3624.720763	350.4332311	-1438.854389	2284.561366	730.1883744	-1630.497794	-457.5806561
2014-08	1595.95064	150.6412335	602.4860258	684.0294043	922.727789	-1462.413731	-600.4247989
2014-09	-3281.738196	-423.8296466	1483.326478	-1137.709991	1015.203095	-1284.421107	-723.8862273
2014-10	-3434.736846	-290.3704118	1134.539438	-2620.683644	966.5844013	-1110.855558	-827.586367
2014-11	-3873.869665	671.9092929	581.6323114	-3344.151819	776.3639185	-954.82469	-911.1739921
2014-12	1552.45771	807.2535711	-1491.952943	-3324.378762	479.1727402	-831.5281043	-974.0753497
2015-01	12023.24204	-764.7514629	-2629.803631	-2543.917939	150.07486	-751.8036469	-1015.969601
2015-02	-12969.78964	-1544.574344	-1834.511536	-1091.65699	-117.1387537	-721.0463853	-1037.088035
2015-03	12206.30285	828.6167879	314.307275	927.1688036	-260.750685	-739.5043585	-1037.882856
2015-04	-2298.392563	1175.225994	2634.362961	2738.670514	-272.0751383	-802.634417	-1018.959332
2015-05	-4101.90128	-205.8076156	1905.204356	3438.665515	-158.1452566	-902.8843996	-981.1844638
2015-06	-4358.609951	-457.5490368	787.0004667	2950.754369	50.33138393	-1029.997619	-925.4727506
2015-07	2259.062198	-93.9092324	-657.1998279	1599.654094	283.2685531	-1168.231565	-852.9463563
2015-08	-1154.629552	-185.9154567	-49.25624037	-34.6166622	491.1960051	-1300.221897	-764.8814906
2015-09	1152.604868	-15.45113443	-637.3030168	-1508.469578	650.4494566	-1410.946723	-662.497805
2015-10	-1547.469348	296.4223685	-524.8885848	-2758.444652	717.5275647	-1493.477208	-546.8752714
2015-11	368.9668121	621.5398309	-1312.838734	-3352.854524	656.100064	-1541.917177	-419.1387148
2015-12	4644.240996	47.69934671	-2622.711031	-2878.628684	466.3588551	-1553.086287	-280.341462
2016-01	3849.070912	-1267.365917	-2130.986493	-1594.783382	206.1359799	-1527.686102	-131.638559
2016-02	-8396.075813	-829.382563	396.7293335	255.1948547	-94.75427564	-1467.594811	25.2961807
2016-03	8110.400346	1878.158927	2858.437322	2138.887903	-388.6118841	-1375.452451	188.6192823
2016-04	-4820.037219	1443.692298	2296.402177	3451.620985	-641.791247	-1252.578802	356.4185076
2016-05	528.6275605	-1271.546434	1136.724014	3702.349508	-824.661811	-1099.234134	526.9661424
2016-06	-2803.746264	-1386.646926	332.4265438	2769.652541	-935.5487623	-914.0115281	698.7832375
2016-07	-2041.720714	136.4855721	490.5421348	1004.532076	-979.4438777	-694.2934546	870.6548423
2016-08	4348.746687	876.893443	1414.22951	-1076.976691	-959.3043396	-436.2392923	1041.403277
2016-09	-3776.653182	302.7061749	907.0648782	-2729.918147	-876.3778515	-138.7772663	1209.849807
2016-10	-2127.77769	-31.99697753	-2168.293816	-3559.079423	-727.5019175	194.9671328	1374.866051
2016-11	4914.271753	-316.2906298	-3675.549742	-3552.881398	-545.427776	559.0340135	1535.461425
2016-12	3264.190465	-727.6432041	-3261.642165	-2767.139492	-369.5632754	946.0444121	1690.949268
2017-01	-6703.406864	-835.4911235	-150.858544	-1238.475561	-238.2068316	1348.222033	1841.141991
2017-02	-641.9945887	438.491074	2626.842238	736.8871635	-159.0570734	1755.33264	1986.314526
2017-03	6342.21596	1605.306425	3389.833117	2470.293146	-88.68363302	2154.79545	2127.106459
2017-04	-5335.841722	300.3833081	2006.034837	3589.555504	2.84198215	2536.020305	2264.065925
2017-05	2125.923705	-1199.014079	-1173.177816	3802.892892	131.7958348	2891.460943	2397.483615
2017-06	364.231792	-901.8666305	-985.8610547	2795.018571	286.1381498	3216.548775	2527.537137
2017-07	-2383.086686	488.8371283	10.67891247	900.5536641	417.1897488	3509.737834	2654.269559
2017-08	3098.920987	968.3794714	1164.998124	-1228.408699	468.9907773	3772.020001	2777.7412
2017-09	-561.7987116	-420.8228187	493.6201239	-3000.938145	434.7573362	4005.27887	2897.995648
2017-10	-4937.327824	-1619.250163	-995.5047174	-3953.179064	321.6799591	4209.774926	3015.277982
2017-11	2391.930149	86.91984052	-1736.650305	-3841.478995	160.7467641	4381.327424	3129.897789
2017-12	-1264.25338	2432.849143	-1981.564519	-2546.15493	-17.56298551	4513.086732	3242.259565

2018-01	11373.28944	-911.3515839	-2094.441521	-371.9181696	-171.9220885	4601.053139	3352.565355
2018-02	-12317.82498	-3306.874092	-799.2855886	2156.672733	-259.9461143	4644.128637	3460.597022
2018-03	11304.49853	1621.06576	2269.354026	4151.836584	-273.1848935	4643.758436	3565.877464
2018-04	-5714.051994	2910.109131	2956.303581	4733.350792	-202.868013	4601.71304	3667.934522
2018-05	2606.496719	-1289.787508	1781.715773	3973.51021	-43.58651752	4518.598307	3766.417034
2018-06	-4655.997642	-1798.216679	244.1991156	2613.785755	241.9759571	4391.902564	3861.233782
2018-07	786.9564024	478.8371374	-654.5488826	704.7899742	653.0609758	4218.595194	3952.41245
2018-08	3106.381167	1282.832858	-339.5014783	-1907.717927	1138.957837	3999.074331	4040.058331
2018-09	-1958.485704	98.18486543	-1783.361232	-4379.135218	1643.634057	3737.93254	4124.129592
2018-10	-1630.365556	-1046.647905	-810.7321419	-5942.535051	2093.825167	3436.782905	4204.281201
2018-11	3467.568536	-88.51122021	-50.80851988	-6251.013027	2397.469894	3096.548305	4280.007498
2018-12	-4978.610196	1706.276216	1107.109941	-5176.039848	2524.016824	2717.158718	4350.976968
2019-01	7527.028838	130.7507859	-215.6160284	-3080.536702	2502.573724	2298.119157	4417.191925
2019-02	-9253.824169	-1548.476579	-289.2578174	-890.1433034	2384.83855	1837.133161	4478.857093
2019-03	9717.152366	278.5087574	235.3268592	1244.150545	2236.938555	1333.581413	4536.023809
2019-04	3609.253501	1085.518671	99.14518599	3601.164163	2034.836954	792.4867645	4588.702878
2019-05	418.4873533	-1.82962861	-1191.119112	6084.779796	1708.578056	222.7070888	4636.724198
2019-06	-7641.835914	-740.4798806	-1973.232973	7536.173493	1216.84211	-368.0523216	4679.856568
2019-07	7611.497359	-666.7620133	-794.789291	7281.437694	520.9467395	-971.7311812	4718.280185
2019-08	267.1088732	149.3945982	2917.176957	6013.414991	-398.1727492	-1576.251997	4752.422106
2019-09	-9866.998631	1050.43828	6297.77423	4216.384129	-1461.879592	-2170.564633	4782.696465
2019-10	-7180.793036	1548.116946	7920.317473	1895.025463	-2548.718894	-2746.472931	4809.634122
2019-11	4678.421162	1955.805776	3589.909814	-1221.629895	-3553.443447	-3298.167509	4833.981028
2019-12	15991.17057	912.3904306	-2210.443027	-4492.047516	-4390.659027	-3819.789164	4856.560647
2020-01	16315.22385	-1515.101825	-8308.258641	-6473.083098	-4987.862156	-4304.030439	4878.191677
2020-02	-17887.45939	-3182.142696	-11207.66435	-6618.290419	-5294.583626	-4743.932734	4899.715794
2020-03	11871.08218	-1549.454638	-7061.3441	-5285.71885	-5274.864376	-5133.804184	4922.083577
2020-04	12888.63967	889.0804534	70.03648415	-2933.340945	-4914.465694	-5469.930412	4946.202629
2020-05	-1884.351422	1814.532217	7062.470262	-269.2095719	-4206.90293	-5749.836758	4972.825975
2020-06	-5471.571278	733.7947716	10443.61863	1718.562668	-3170.562225	-5976.20887	5002.431948
2020-07	1247.553556	-778.7095571	9616.636678	2870.756615	-1873.196834	-6151.523624	5035.464755
2020-08	-1828.417197	-409.5146889	6725.526564	3149.084293	-427.3116609	-6276.355568	5072.370966
2020-09	2543.056067	155.1465392	3917.1271	2516.046705	1052.416452	-6351.235574	5113.812903
2020-10	-3978.280403	40.37484774	112.3849444	1599.901588	2512.471833	-6378.617586	5160.823811
2020-11	4149.04609	618.7977148	-4477.316377	831.8166032	3890.072653	-6364.430844	5214.588147
2020-12	5874.459378	579.0784376	-7262.368955	123.234595	5099.594106	-6315.114137	5276.158126
2021-01	5507.909007	-1406.90314	-7768.402681	-217.5934266	6062.110986	-6237.042368	5346.513657
2021-02	-13092.03643	-1564.769549	-5559.33575	113.6184172	6718.303092	-6135.917748	5426.501326
2021-03	13454.01526	1151.061301	20.97921327	955.4107324	7097.546514	-6018.20519	5516.688099
2021-04	-354.8708639	1205.417843	3502.758307	1638.850723	7280.878473	-5889.52097	5617.62386
2021-05	-5272.628215	-316.0852834	4401.573736	1733.774115	7349.872374	-5755.941581	5729.885148
2021-06	-7470.597354	-591.8139143	3288.389801	1166.471937	7342.045616	-5616.888529	5854.002698
2021-07	5280.838089	141.1369859	1312.275666	-9.958769008	7241.488913	-5469.497247	5990.509805
2021-08	-1368.011729	637.8762132	-1403.755874	-1576.894156	7003.701986	-5311.040338	6140.287451

2021-09	1100.696169	-183.8598654	-1964.023249	-3071.881342	6577.26272	-5138.376327	6304.052257
2021-10	-3118.972354	-505.6325549	-2087.940829	-3981.194757	5885.583227	-4947.132348	6482.34669
2021-11	1317.07993	86.47747624	-352.3930996	-3633.76376	4886.219119	-4732.949377	6675.201723
2021-12	3924.9222	524.6163688	1135.565876	-1884.251346	3577.957106	-4492.577507	6882.234652
2022-01	4462.7429	-79.82655185	1018.280115	622.1173356	2008.688541	-4227.858326	7102.83524
2022-02	-8223.255915	-212.5407925	-325.9584132	3162.711363	237.7741268	-3946.358005	7336.162614
2022-03	8830.635496	358.5576218	-1596.680536	5210.528882	-1617.559673	-3655.410742	7581.180499
2022-04	-8450.951696	-121.4696852	-2120.578435	6606.022775	-3481.996276	-3358.217032	7836.401887
2022-05	-1469.625176	-839.6847658	-569.338361	6879.954344	-5290.76474	-3053.326158	8100.27034
2022-06	551.5177796	-223.6484122	2692.997231	5929.354625	-7021.289975	-2737.970107	8371.153916
2022-07	4499.393179	1101.366714	6046.317427	4014.694748	-8661.444058	-2411.599792	8647.411157
2022-08	8677.724551	2283.551847	6716.833165	1486.453169	-10181.24411	-2074.307691	8927.609442
2022-09	1694.495108	2213.079322	3012.356151	-1528.754264	-11458.90825	-1723.049798	9210.413589
2022-10	1877.658872	-37.56818437	-2699.564576	-4723.452026	-12332.79602	-1350.618871	9494.591707
2022-11	8558.925741	-3661.572044	-7398.662858	-7221.503525	-12641.41272	-952.6499267	9779.426635
2022-12	-16294.23213	-5020.648954	-8343.562801	-8145.319982	-12261.41167	-529.8821804	10064.65789
2023-01	-10575.39341	-2155.562617	-4653.672833	-7028.632456	-11211.28385	-82.10892736	10350.112
2023-02	15381.41627	2120.78626	1107.36281	-4217.337343	-9616.662139	389.1284818	10635.49735
2023-03	13729.85976	4271.316151	4975.615348	-598.4794503	-7622.888709	878.8997294	10920.55496
2023-04	-2046.421224	2725.432373	5141.750377	2862.3006	-5358.601003	1381.042443	11205.08075
2023-05	-2597.387263	-1258.566596	3469.35738	5244.142134	-2901.586248	1889.594869	11488.81466
2023-06	-10771.09139	-2159.129368	2146.532263	6269.590507	-301.3942264	2399.259862	11771.50782
2023-07	-810.2613444	-142.6488335	1787.560345	6118.219689	2394.080867	2902.563274	12052.82664
2023-08	8446.662425	1183.145236	2558.035168	4970.634244	5074.31787	3392.522019	12332.28714
2023-09	-7020.235701	218.5021764	2340.75279	2829.210546	7634.215292	3864.383891	12609.27877
2023-10	946.948094	-443.7886042	119.1567953	153.9018319	9948.857182	4314.786298	12883.03155
2023-11	5034.489184	207.6268151	-2141.676997	-1831.485995	11890.94649	4742.67937	13152.42201
2023-12	-6675.335904	625.6877345	-3258.280782	-2545.209988	13397.17235	5145.176348	13416.26319
2024-01	8398.868371	-1905.654298	-3210.135862	-2505.726943	14504.81316	5515.654062	13673.53566
2024-02	-9864.361011	-1825.150616	-207.4923395	-1843.321172	15260.2535	5845.513547	13923.25158
2024-03	10128.96164	2853.629143	2865.306418	-504.2546327	15752.2204	6127.845178	14164.84481
2024-04	-4795.025947	2445.734836	3717.994535	774.3803432	15997.64975	6362.202333	14398.05388
2024-05	-201.9964427	-1590.866776	1475.52155	960.9790215	15958.98734	6552.984541	14622.43759
2024-06	-8426.068378	-1416.102139	-271.3269878	376.0427439	15590.8525	6705.887991	14837.47393
2024-07	8133.016427	649.9480683	-1935.967673	45.31121907	14869.77801	6824.50471	15042.57217
2024-08	3803.826633	620.0691211	-2528.012916	195.6234655	13829.82924	6911.898748	15237.05713
2024-09	-8332.937944	-399.4578529	-2978.179987	547.8635535	12493.46303	6968.398861	15420.02922
2024-10	-4992.900129	-775.1529109	-2293.965661	1260.009064	10832.97321	6995.430343	15590.47318
2024-11	-3375.771262	-62.24268953	585.5875126	2298.76252	8817.961263	6994.676133	15747.43686
2024-12	2868.851014	701.9550796	3477.941371	2983.827869	6398.110954	6969.175252	15890.01741
2025-01	9249.688359	936.4450843	3382.226625	2677.59555	3584.478373	6921.70723	16017.20497
2025-02	-8577.837535	53.95368593	1841.286655	1626.568649	473.8087316	6852.068112	16128.03815
2025-03	7348.042335	-323.3604962	-875.4815021	213.8929677	-2759.863923	6755.763463	16221.57797
2025-04	-1012.581568	-213.6821317	-2943.948084	-1299.5585	-5943.540845	6628.79774	16296.96501

2025-05	-2674.363746	-663.9271827	-2311.070222	-2484.672235	-8955.757304	6472.78713	16353.56413
2025-06	-4079.941583	-243.7550903	-1075.964519	-3106.254928	-11700.71147	6296.932345	16390.61316
2025-07	4219.615492	490.8370306	1065.187276	-3222.634646	-14087.01643	6112.092756	16407.45166
2025-08	746.1636464	469.366634	1979.872453	-2773.711866	-16029.60758	5929.78072	16403.53535
2025-09	315.0436751	-178.3316909	269.6750359	-1843.198421	-17438.9343	5764.836761	16378.39086
2025-10	-4152.171194	-651.3051092	-1044.179584	-942.1529405	-18224.4571	5633.47685	16331.50431
2025-11	-3084.396822	-400.1580295	-510.0113418	-509.4246149	-18316.55108	5551.264087	16262.28792
2025-12	3068.104173	618.0904209	-324.4384099	-521.1401378	-17703.98323	5525.966909	16170.69315

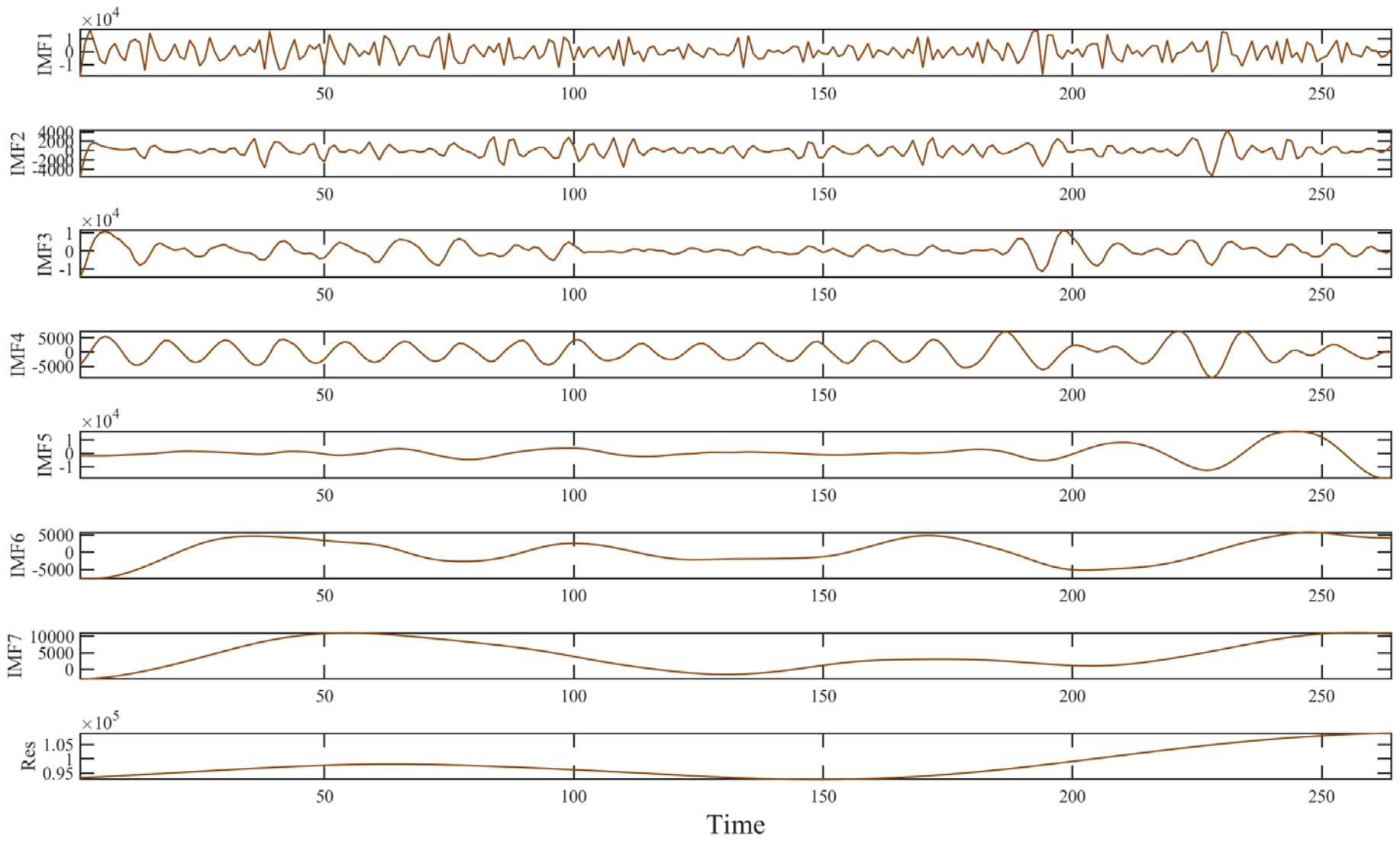

Supplementary Figure 2. Data decomposition results based on the CEEMDAN method.

The fitting effects of the prediction models

Supplementary Table 4 reports the fit results for the GRU, CNN, SVM, and Transformer models. In accordance with the input–output window construction rule, the first 12 observations were used solely to build the inputs and could not serve as targets, so no fitted values are available for those initial 12 original data points for any model.

Supplementary Table 4. The fitting effects of the models

Time	Raw data	GRU fitted	CNN fitted	SVM fitted	Transformer fitted
2004-01	36301	/	/	/	/
2004-02	75937	/	/	/	/
2004-03	100375	/	/	/	/
2004-04	98138	/	/	/	/
2004-05	90822	/	/	/	/
2004-06	89361	/	/	/	/
2004-07	100026	/	/	/	/
2004-08	101221	/	/	/	/
2004-09	89089	/	/	/	/

2004-10	80007	/	/	/	/
2004-11	91169	/	/	/	/
2004-12	87203	/	/	/	/
2005-01	81693	77376.3513	74201.4874	60864.7080	63788.8615
2005-02	62794	63361.0380	73360.2064	71815.6652	69444.0515
2005-03	101475	100635.8081	96368.4062	97456.2312	94347.6603
2005-04	98758	100588.0070	99461.4616	94695.5335	96354.0081
2005-05	94318	93796.6929	96939.9161	92627.0950	95144.5836
2005-06	94136	90752.1252	92939.9848	95677.3390	96704.1142
2005-07	98588	100949.7980	102124.8224	104019.1680	104696.1041
2005-08	105752	103973.8127	106072.6886	106824.9790	107634.0573
2005-09	96004	100124.0162	96029.5996	97154.5152	98360.3384
2005-10	94489	98229.6315	94509.4382	94980.3912	95452.5115
2005-11	106598	106517.6654	101792.1054	105556.9662	105214.9807
2005-12	98200	99602.5618	100866.4413	98704.6511	100128.4426
2006-01	84489	91550.5822	94086.0323	95238.3406	95051.8672
2006-02	92876	100223.0149	93000.7415	94967.5842	96526.4871
2006-03	116831	111065.8123	114683.1879	116096.3011	116284.3567
2006-04	109330	112137.8764	108982.6789	108916.8522	111483.0278
2006-05	104575	109309.2752	109530.7690	106000.4340	108005.8598
2006-06	108312	107099.6722	102998.9953	107976.0431	109865.8371
2006-07	111905	113313.9341	117482.5296	115385.0688	117597.6823
2006-08	115960	118194.7259	115231.2613	116104.1468	118198.1972
2006-09	103665	107500.2355	103947.9953	102182.6453	103363.7072
2006-10	102399	99191.7483	104925.6266	98809.1880	100004.3633
2006-11	108022	109024.6295	108607.2307	110112.0972	110269.7975
2006-12	103371	106105.4354	107212.8726	105587.1925	107095.1506
2007-01	106956	101353.7879	97885.0472	93001.7991	96187.3616
2007-02	83225	88100.1014	98436.0852	88976.3163	89598.0554
2007-03	122566	120137.5097	112010.3756	114315.5262	113160.9027
2007-04	114959	115929.3414	114483.1537	109575.8094	111398.8879
2007-05	108239	114186.1155	117078.6533	113930.2746	116026.7219
2007-06	110126	115574.0474	113425.4971	122185.5278	122881.4828
2007-07	119639	122312.8714	120830.0571	125316.6265	126068.2556
2007-08	124674	123282.6606	119138.3534	120624.9020	122291.7049
2007-09	106206	110987.7690	109488.0432	106378.7420	107485.5637
2007-10	106364	105478.5320	107418.7866	106271.4723	105841.4006
2007-11	114606	113248.2601	109789.4438	114006.7377	114624.2781
2007-12	109665	111679.0822	108544.5394	108476.4132	110910.7882
2008-01	108255	106167.7223	105383.1650	106962.0735	105890.0903
2008-02	90471	90185.6327	99054.0470	91812.6949	92462.2153
2008-03	123200	121881.9360	119188.0216	121080.0829	117944.4191
2008-04	118789	117132.3436	116148.6198	112181.3975	112879.7573
2008-05	113200	111132.3608	111568.2394	108107.9766	109538.5534

2008-06	107306	107200.9415	110372.1731	109364.5399	109588.4143
2008-07	123352	118516.3338	117762.7976	119454.5834	118715.0822
2008-08	115787	119218.1327	119745.3640	117074.9890	116738.6580
2008-09	106026	108106.2466	106644.6096	105694.3038	106608.6217
2008-10	108840	101504.0711	103404.9747	103532.9774	103158.7769
2008-11	105265	108023.2601	112036.4876	109417.8476	109716.9987
2008-12	110163	110033.4007	109635.7396	106981.3183	108651.7865
2009-01	90569	100144.0747	101803.5753	99619.6820	99671.3855
2009-02	120974	111883.9417	104827.4322	104408.6003	104611.5235
2009-03	125427	120253.5701	121218.0832	118432.8700	116879.5439
2009-04	120701	111967.6039	118948.3170	115658.4145	115641.8690
2009-05	110617	108898.4668	110527.9752	113776.8434	113144.7089
2009-06	116439	117914.5196	118899.9361	117675.7332	117079.2460
2009-07	123255	123609.3471	118667.8269	126314.5957	124419.1907
2009-08	119965	119054.9407	111308.7732	115289.6091	114715.2566
2009-09	107161	105168.0079	104799.5462	100943.6444	99198.8794
2009-10	100504	99286.9027	101217.7681	103084.7634	101290.6881
2009-11	93627	96133.7314	105349.6459	102631.8884	102890.2825
2009-12	101113	102470.4672	95126.5571	103897.6729	103132.7709
2010-01	102470	94890.2658	96830.0549	85401.7938	86205.6819
2010-02	80143	85948.6996	95734.5362	95665.5247	92240.1806
2010-03	117048	114772.8065	105403.3956	109950.4453	107688.4849
2010-04	104691	105997.2381	110271.6080	105371.6910	106392.0496
2010-05	100653	103134.4977	103345.6961	104790.3917	107155.4479
2010-06	98691	101259.2731	101635.3108	107307.1383	107459.1939
2010-07	105007	106839.0534	101149.1567	108907.1628	108008.3799
2010-08	103030	103758.3348	105897.3110	103511.5261	103634.2218
2010-09	93643	92498.1846	96690.3425	93875.1978	94415.7869
2010-10	89066	89580.5626	89589.8340	88970.5016	88708.2991
2010-11	101234	98529.9695	99490.2414	96372.4133	96608.2189
2010-12	97590	102775.2403	100500.8321	103147.8694	102768.7328
2011-01	100932	100706.0003	97161.7626	101446.9114	100141.5940
2011-02	85649	86065.6758	95418.3605	85497.3380	86268.7295
2011-03	117553	117945.1518	113459.0549	116633.4550	112763.1542
2011-04	106464	110138.4192	111798.9283	108740.9965	108746.2874
2011-05	106263	106760.9435	104238.9287	107847.7634	108389.4450
2011-06	103219	104152.0264	104490.8556	105354.4358	104850.1650
2011-07	108259	109605.2398	109423.9522	112537.5582	110469.8319
2011-08	113093	112397.6711	111491.3534	112438.4402	111030.6000
2011-09	100517	104290.9240	102339.6446	105732.9217	105003.6665
2011-10	99591	102920.7252	100503.5615	102328.2020	101392.2730
2011-11	107208	105956.2624	100085.1149	104701.3049	103894.8986
2011-12	103488	98234.0844	100946.7174	95940.3448	95863.8522
2012-01	89614	94674.9898	100903.3638	101659.1658	99159.2242

2012-02	123239	115093.1801	103832.6997	109680.9665	109095.3516
2012-03	124899	120420.0525	123529.1662	120230.5098	118792.9603
2012-04	107415	108534.0139	109037.4714	109841.8196	110363.9898
2012-05	113147	106946.4958	104404.7914	108710.0613	106783.9744
2012-06	101592	98363.9361	104542.1783	99980.3531	99081.6664
2012-07	108079	104774.0502	105645.4884	109132.2243	108305.5842
2012-08	107139	105591.3308	108631.4455	106654.4367	104985.5419
2012-09	94868	96256.2792	91937.3737	92686.3639	92177.4098
2012-10	95678	95802.3286	98573.9853	95381.9905	94771.2611
2012-11	100209	97580.5796	101693.5825	102304.8264	100413.3270
2012-12	91441	95471.3132	92488.8842	99576.3585	98052.2024
2013-01	102367	98363.7481	94104.7515	92784.1369	93959.6818
2013-02	78884	85884.2752	92231.4035	93409.9577	91732.3353
2013-03	107535	103750.9094	96856.7196	103294.7564	101518.5692
2013-04	97225	94550.2940	98519.9178	93726.9638	94918.2427
2013-05	96978	95271.9069	94758.9786	99956.7697	98857.0305
2013-06	86335	88225.6102	91975.5920	89924.9219	90750.7200
2013-07	97401	97277.3489	95597.4633	99136.3714	97798.3783
2013-08	97430	94334.3439	94512.9416	94789.5012	94015.1507
2013-09	87915	87294.5537	89642.1239	90103.6450	90208.3377
2013-10	86161	83899.8942	88746.0657	88877.9591	87857.4007
2013-11	88479	88219.1481	88847.7332	92548.9028	91835.9501
2013-12	87609	89396.2770	91943.1577	86577.3353	87052.4897
2014-01	90210	90762.9584	88967.3394	93086.1577	91184.8297
2014-02	83068	82532.9239	86571.7051	82693.1140	82942.0527
2014-03	99292	100610.5938	99861.6012	101482.1408	99077.1416
2014-04	94768	92833.9951	96364.2604	94079.2817	94441.6400
2014-05	91936	90558.5516	91942.6105	96091.1311	95768.0097
2014-06	88201	89046.5104	91479.0875	91095.4811	91028.5095
2014-07	95648	96853.6342	95140.6812	99567.4640	97566.6205
2014-08	94075	93879.0344	95880.1652	94953.6195	94705.0099
2014-09	87827	88528.8249	87988.9464	89698.1332	89570.9025
2014-10	85996	86945.8509	87975.7813	89237.3234	88720.3448
2014-11	85125	90678.7255	90174.9889	92847.8180	92380.4229
2014-12	88397	92225.5661	89713.4218	93564.1140	93452.5344
2015-01	96649	88200.4765	86940.1335	89100.8101	89544.9073
2015-02	72869	78995.8023	85117.5776	77718.6459	78578.1645
2015-03	104427	103104.5258	94597.6894	98411.2138	96556.9531
2015-04	94350	94311.4354	94907.1048	93387.1144	94875.8194
2015-05	91194	93571.9370	91210.2806	96108.2857	97295.9054
2015-06	89224	90278.1658	90416.9037	92555.8403	93305.3396
2015-07	93586	94500.5037	92532.8132	95502.3656	94673.6676
2015-08	89228	91579.9342	95658.1934	91918.4957	92133.8711
2015-09	89806	88826.8000	87976.7672	89579.9665	90801.2182

2015-10	86393	85853.0104	86033.0102	84908.4596	85281.4643
2015-11	87284	87147.2860	85439.2439	87485.4939	89046.1691
2015-12	90103	90431.2695	88517.9721	87359.3563	89129.4792
2016-01	89699	87779.9572	89573.0885	88380.7089	88138.6417
2016-02	82204	82616.5688	90302.4326	81847.3301	83741.9325
2016-03	105745	106595.8587	101760.4173	105908.0211	104846.2660
2016-04	93190	97472.7642	100594.8141	95453.3352	97418.5156
2016-05	95079	94102.9106	91308.4838	95559.2215	96217.8709
2016-06	90166	87551.3407	89909.5459	90332.9823	90401.3958
2016-07	91219	92257.1222	92497.6675	93595.8388	92868.0427
2016-08	97670	93874.9763	93440.2596	93647.0823	94277.7526
2016-09	87390	88536.6230	90629.2831	91253.5596	92049.8126
2016-10	85480	87117.3089	85724.4427	87583.5928	88869.7400
2016-11	91478	89938.4101	86467.4496	88630.2272	90546.5778
2016-12	91371	88755.0289	92604.5760	88426.4832	89697.1726
2017-01	86657	89151.5694	91492.6979	92017.1396	92896.5875
2017-02	99417	100142.9892	98061.0869	97364.5746	99371.6759
2017-03	110717	108334.4999	109863.8971	107821.5446	108309.3271
2017-04	98123	103154.2317	103954.0931	100881.7703	102513.8188
2017-05	101783	102544.8323	102808.7795	102756.0319	102885.2276
2017-06	100155	94319.9322	96838.3915	96701.2865	96871.6357
2017-07	98501	98446.7908	101322.2024	101102.8114	100554.6378
2017-08	103977	105037.9001	104111.3627	104587.6170	104963.3481
2017-09	96856	95756.1262	96197.6620	94476.3509	95800.1577
2017-10	89105	92090.0028	99234.8299	95215.1760	95398.4277
2017-11	97694	101016.4723	97728.1857	101696.7225	100840.7863
2017-12	97560	98581.6406	98626.1500	100646.2911	100020.3662
2018-01	109021	100362.0275	97750.9478	94895.8262	96956.9553
2018-02	86886	93094.3084	100451.0928	94224.4455	94164.1468
2018-03	120659	115784.9742	109422.1097	113319.6349	111656.5582
2018-04	106398	108659.8989	109480.4474	106756.8402	108955.5913
2018-05	108831	110050.7884	108041.5866	110593.7592	111113.2188
2018-06	98491	97874.0708	99642.1334	103049.0817	103163.3691
2018-07	103809	101534.1370	104984.3729	102455.5561	102799.9882
2018-08	105068	101686.1193	107273.3655	103603.6785	103847.2317
2018-09	95312	96330.8692	96202.0865	96875.2272	98636.2365
2018-10	94217	94583.1423	96898.8534	91916.0338	92984.8519
2018-11	100849	100967.0864	95517.8157	99965.8586	100320.8947
2018-12	96336	100269.9689	98866.8080	100191.2570	101572.9629
2019-01	107754	108317.8828	100256.7792	109404.6016	108045.6831
2019-02	90985	93615.3869	97261.5849	92274.0057	94860.8306
2019-03	113941	113760.5773	106288.9094	112053.1223	110693.1813
2019-04	110266	104199.7184	105532.4834	104235.9346	104783.8277
2019-05	106431	100256.0331	106283.1966	105577.1250	106211.4673

2019-06	97362	97023.2279	103994.4582	100778.0261	100856.3289
2019-07	112454	107518.6751	110073.3033	107761.7196	106449.8352
2019-08	106985	106349.0640	110468.5609	105829.0574	105109.0765
2019-09	97815	104550.6038	100911.0071	104236.8061	103605.6995
2019-10	98774	100618.8592	99361.8725	104550.4244	103650.1088
2019-11	102174	100209.6467	95079.6759	101886.0393	101616.3721
2019-12	102151	91732.9251	88446.0402	86312.6821	87884.9690
2020-01	91026	77903.3628	77919.7667	73801.1355	72993.7586
2020-02	51506	54365.5327	68157.7793	56606.4512	57251.2117
2020-03	88150	90332.3100	82399.2981	83474.7104	81947.7155
2020-04	101262	97642.5023	90395.4016	86269.6419	87503.5738
2020-05	97651	96520.0380	88800.6862	89221.6701	90220.3521
2020-06	99319	96698.8063	97779.7598	91545.3083	91255.6055
2020-07	106135	104617.6940	105710.1292	104615.1696	103187.5288
2020-08	102304	101890.8706	104711.8220	103107.2554	105070.2611
2020-09	105377	104147.2510	104694.2439	97915.2650	99524.3395
2020-10	95633	98957.0742	91336.6093	91654.4864	90228.8153
2020-11	100561	102592.8773	99576.6347	98496.1625	96649.6235
2020-12	100209	100003.0841	101949.1646	99482.5475	98269.3518
2021-01	98257	95711.8548	96769.3833	95431.8889	93622.9211
2021-02	83014	88730.4059	96507.7440	83211.8672	84414.3582
2021-03	119423	117804.4793	105364.2331	114873.3270	112766.6910
2021-04	110385	116207.4632	115989.6238	113543.6711	113291.8765
2021-05	105393	106139.9193	107523.1199	110363.4000	110905.1465
2021-06	101633	101377.0540	103193.5597	106354.3405	107014.8157
2021-07	112287	107928.2536	108956.7828	110696.3379	111117.6619
2021-08	102061	108137.6689	109063.4013	103323.2755	105752.2416
2021-09	101701	103682.4284	100055.0266	101307.7828	102678.3959
2021-10	95942	94005.4489	96778.5669	92433.5752	94048.4517
2021-11	102598	105260.6634	108258.1410	103677.7824	105811.1937
2021-12	108157	106951.1084	107888.2516	106327.4562	108538.6180
2022-01	109531	103367.9940	101622.2642	104408.0774	105739.3404
2022-02	96787	96884.5720	101122.0689	94245.4293	97520.4605
2022-03	114003	116467.7478	113993.7209	114300.9904	113557.9006
2022-04	95933	103425.5012	109030.2477	102064.2481	103732.4900
2022-05	102912	103768.2686	102392.0779	104329.1482	105539.2663
2022-06	106846	101015.1184	102571.8956	103414.5515	104528.9088
2022-07	112648	105550.3745	108927.1924	109058.9709	109764.0464
2022-08	115375	103702.5464	104753.6942	103308.4872	104698.6599
2022-09	101083	92609.8406	97489.6361	95416.2743	96274.8794
2022-10	90015	84040.0619	89174.6081	85163.4885	86250.8309
2022-11	86371	85610.0502	85239.1070	81505.8318	81995.5601
2022-12	59498	81115.1794	83962.7031	75776.5575	76267.8972
2023-01	74790	101266.6956	84860.8725	92490.2479	90935.8861

2023-02	116063	103591.5465	96530.9392	101608.3968	103245.5718
2023-03	126932	114668.0196	107255.2025	108675.1395	110830.5826
2023-04	116400	110870.0782	111497.4268	102504.0063	105487.0841
2023-05	115934	111073.4998	112321.1468	111821.6382	112693.8029
2023-06	110063	115562.6144	130247.7998	119331.7245	120882.9467
2023-07	125116	135043.8313	129270.9912	134939.4880	135428.3475
2023-08	138875	132652.6271	138124.4981	136382.7141	134510.8641
2023-09	123495	121719.9563	122937.4670	123947.5115	122836.8114
2023-10	129041	130251.6437	130833.8934	128902.9700	127220.0598
2023-11	132270	118264.0605	133533.4772	130772.8567	129913.3207
2023-12	121415	122640.5425	123385.1540	118023.0990	120903.3027
2024-01	135873	125466.4760	130736.4003	127384.3029	128212.0674
2024-02	122780	116459.3087	133494.8377	133836.8995	132274.3778
2024-03	152967	142690.3544	144290.0578	149443.2822	147085.7959
2024-04	140564	135365.1224	141340.7600	139718.6271	142287.9557
2024-05	139523	129571.2808	137777.8021	137122.8976	139785.8470
2024-06	129221	124878.1215	134681.3551	130464.1363	133054.7590
2024-07	145530	133254.8536	138198.4748	138609.5856	140737.6111
2024-08	140045	130358.7940	136782.9876	134026.5490	136056.3664
2024-09	125765	120664.8633	127599.0039	123958.1966	128503.9468
2024-10	128731	126291.2473	130584.4586	131493.2283	133403.6181
2024-11	133186	135444.7900	133172.7502	138117.1089	140757.7822
2024-12	141532	138230.2649	135533.4401	135108.4357	139845.1251
2025-01	145071	137186.7063	129902.2373	136816.9525	138036.2236
2025-02	120756	118317.8309	122964.2600	119119.1766	121645.7054
2025-03	128992	128208.5130	130032.7447	128712.2817	128313.5190
2025-04	113974	112935.7921	122966.4137	111529.5473	114264.5239
2025-05	108245	110351.5213	108819.6541	109531.3919	110768.6842
2025-06	105033	106625.1099	110130.0409	103043.5239	104355.2088
2025-07	113578	115747.6986	112320.8993	113588.8975	112304.6415
2025-08	109355	112733.5739	112033.2558	110038.7985	110366.2497
2025-09	105931	107667.2307	107041.6491	102363.4300	104276.6639
2025-10	99645	107268.6126	103221.3886	99591.1155	100038.8289
2025-11	101715	112166.8956	107111.8984	105973.8617	105649.4847
2025-12	109580	119502.3188	112175.3130	112846.9438	112035.4588

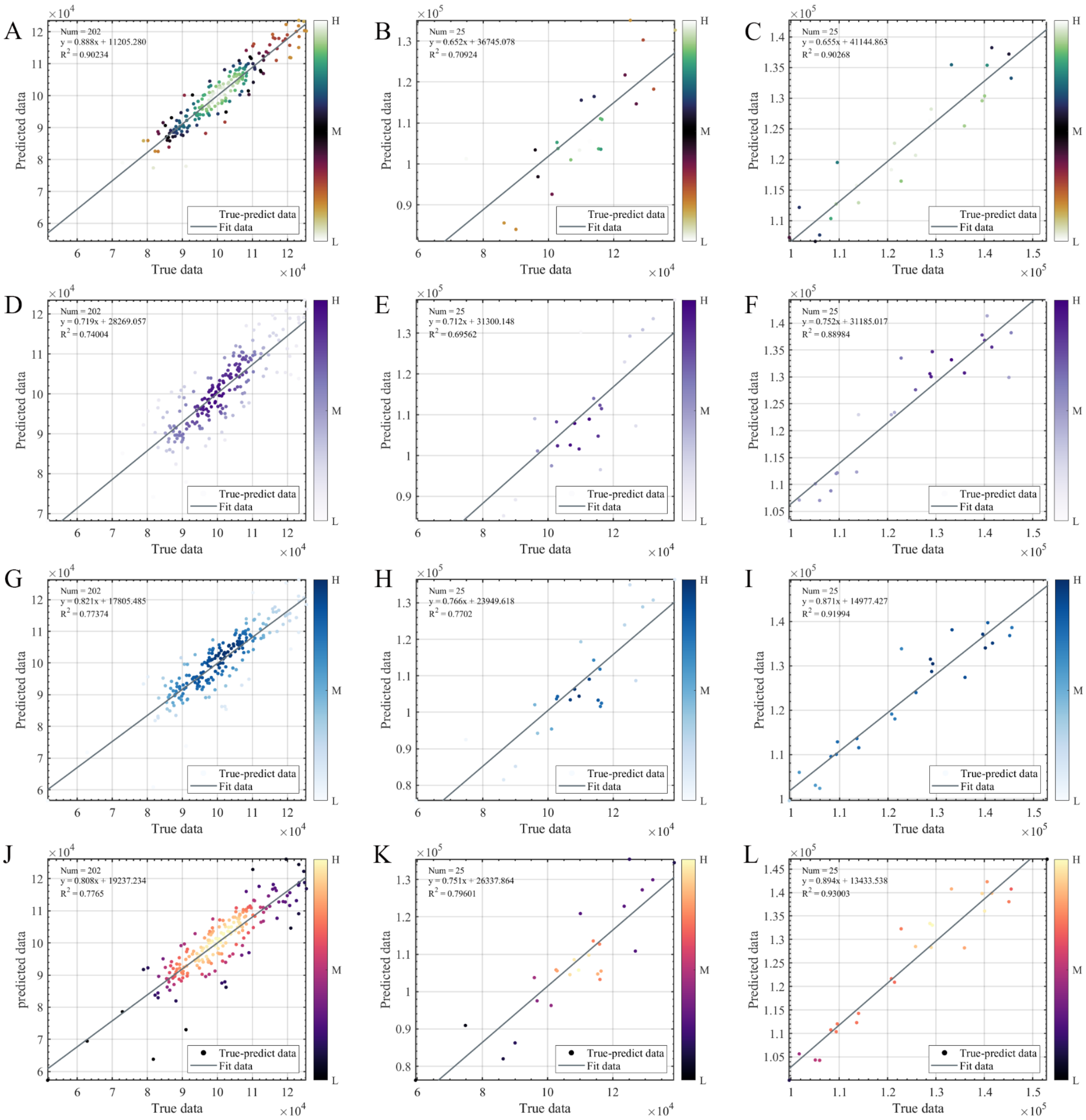

Supplementary Figure 3. The linear regression equations and scatter plots of the true values and fitted values for the training, validation, and test set of all models. Notes: A–C for the GRU, D–F for the CNN, G–I for the SVM, and J–L for the Transformer.

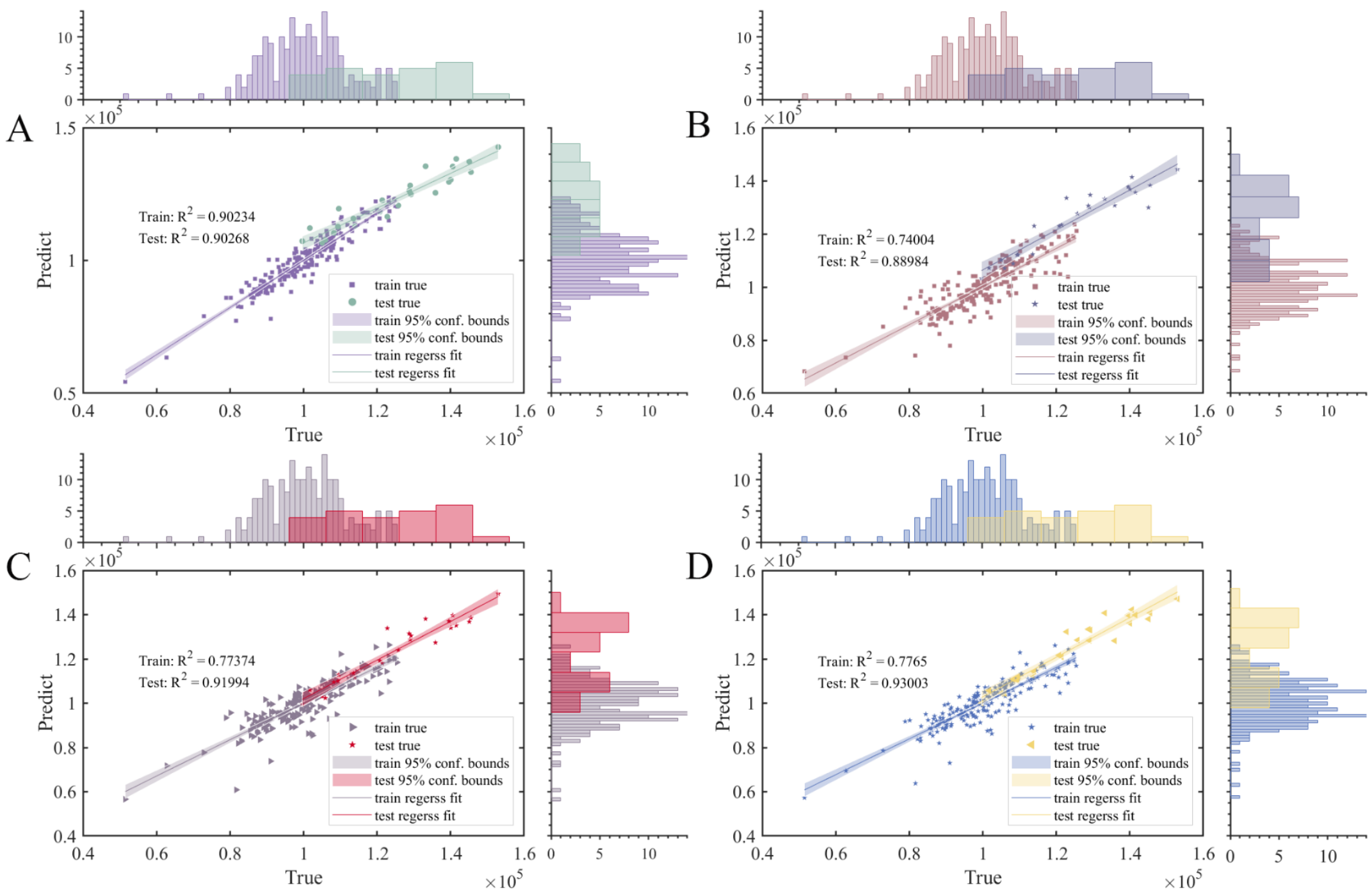

Supplementary Figure 4. The linear regression results of the true values and fitted values of the training sets and test sets of all models. Notes: panels provide detailed scatter plots for each model, integrating separate regression lines for training and test sets, 95% confidence intervals (CI), and marginal histograms.

Comprehensive Residual Analysis

To critically evaluate the calibration and robustness of the four predictive architectures, we conducted a detailed visual diagnostic of the forecast residuals. The analysis includes the temporal distribution of prediction errors and the evaluation of residual autocorrelation, as presented in **Supplementary Figures 5 and 6**.

The bar charts illustrate the magnitude and direction of the prediction errors over the entire evaluation period. While the baseline error fluctuations remained relatively stable across all architectures, distinct temporal clustering of macroscopic errors is clearly observable. Specifically, the most prominent residual spikes correspond directly to major external disruptions rather than gradual epidemiological shifts. Massive structural deviations are visible during the initial onset of the COVID-19 pandemic in early 2020 and the subsequent healthcare policy transitions in late 2022 (**Supplementary Figure 5**).

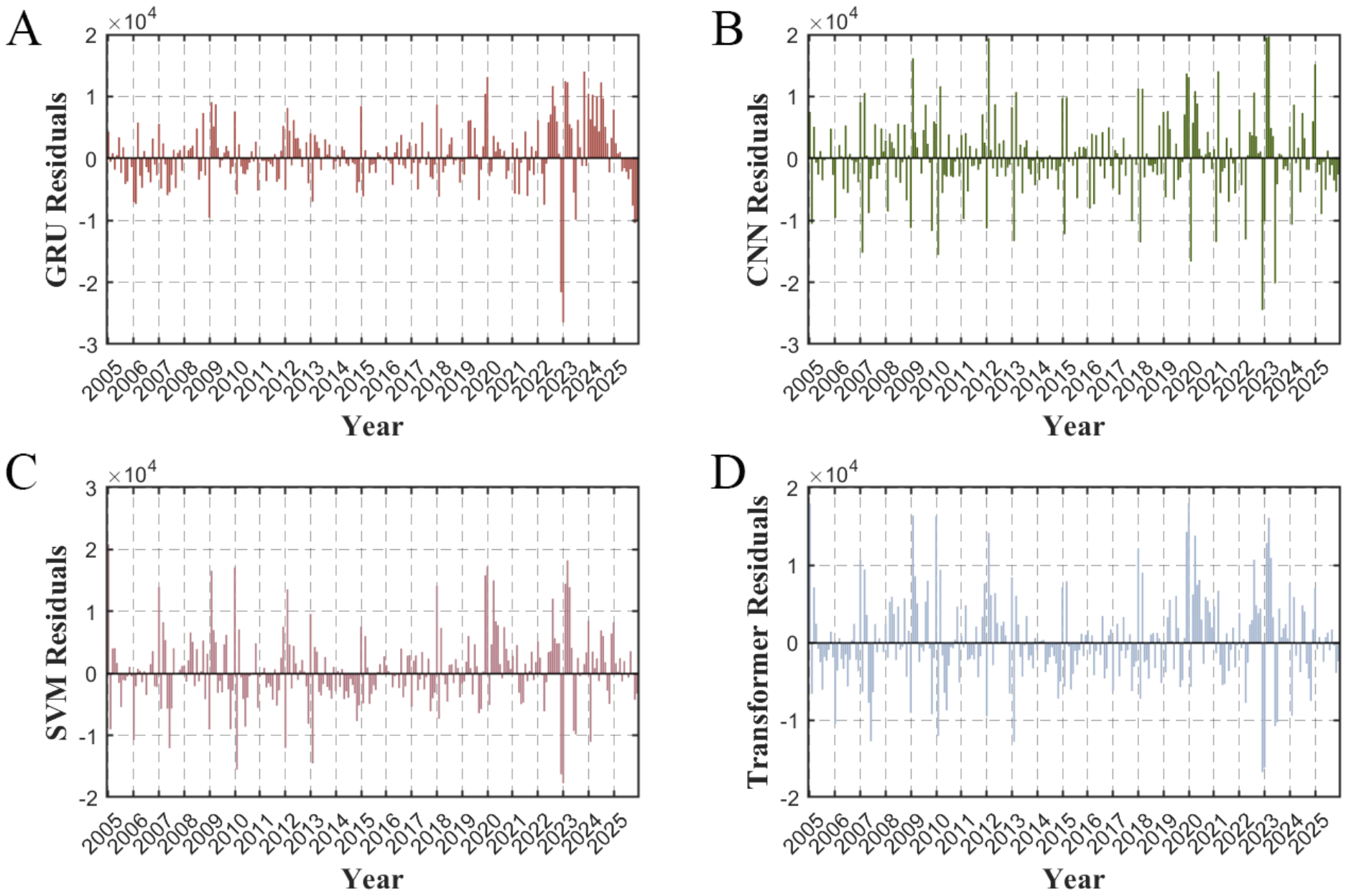

Supplementary Figure 5. Temporal distribution of forecast residuals for the GRU (A), CNN (B), SVM (C), and Transformer (D) models.

The stem plots display the sample autocorrelation coefficients across a lag period of up to 40 months. The horizontal lines denote the 95% confidence bounds. A rigorously calibrated time series forecasting model should yield residuals that resemble white noise, characterized by autocorrelations near zero. As demonstrated in the plots, although the residual autocorrelation coefficients for each model occasionally exceed the confidence bounds at a limited number of specific lag orders, the vast majority remain strictly within these statistical limits. These isolated deviations are likely attributable to the unmeasured influence of external confounding factors rather than algorithmic deficiencies. This diagnostic confirms that the hybrid modeling framework effectively captured and isolated the systematic temporal cyclical patterns from the surveillance data (**Supplementary Figure 6**).

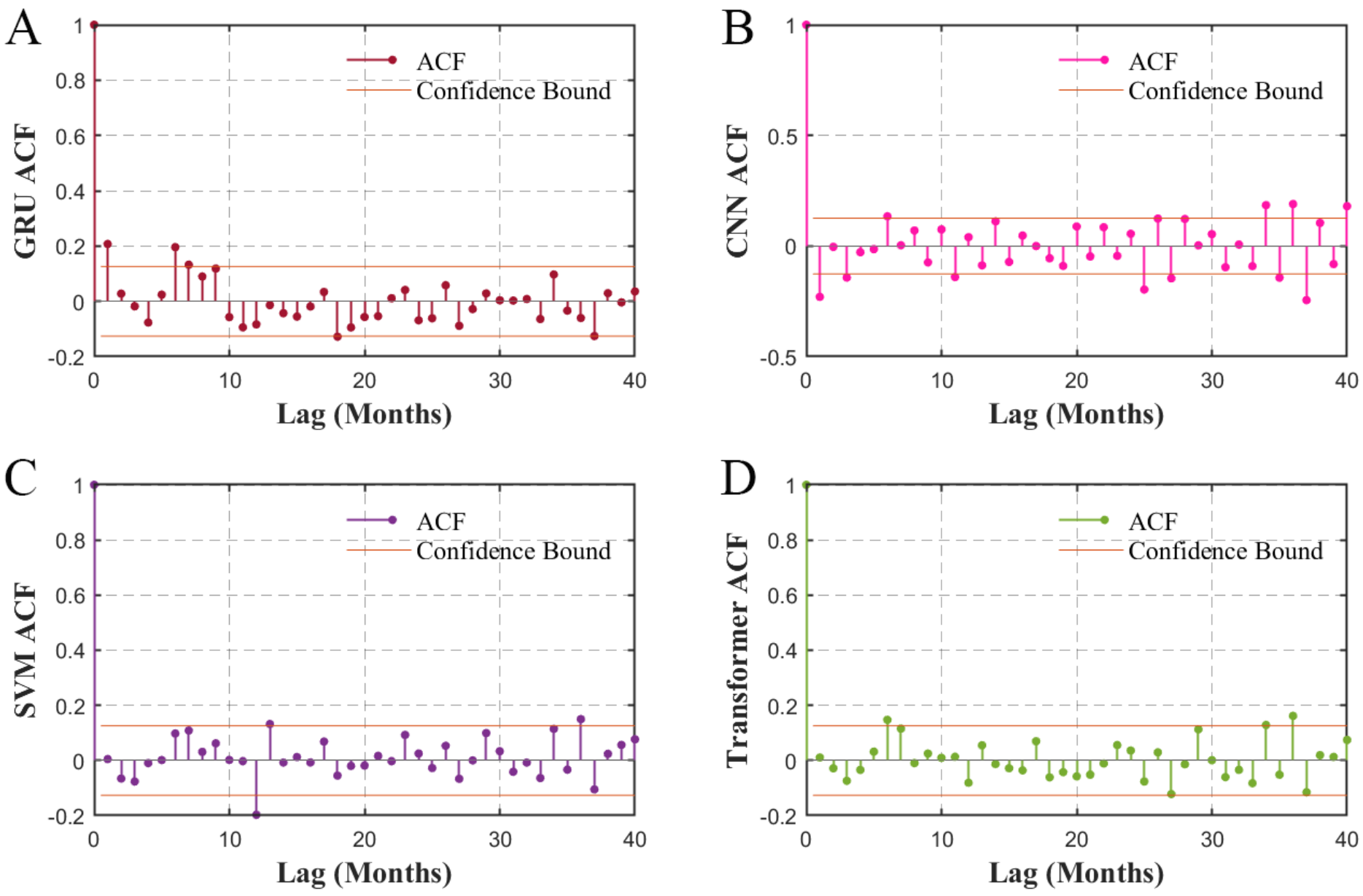

Supplementary Figure 6. Autocorrelation function of the forecast residuals for the GRU (A), CNN (B), SVM (C), and Transformer (D) models.

Sensitivity Analysis

This section presents the comprehensive quantitative metrics and visual diagnostics derived from the sensitivity analysis. For this evaluation, the anomalous reporting data corresponding to the initial COVID-19 outbreak (February 2020) and the subsequent policy transition (December 2022) were systematically removed and reconstructed utilizing cubic spline interpolation. Specifically, the raw observational counts of 51,506 in February 2020 and 59,498 in December 2022 were mathematically corrected to 83,446.76 and 70,914.34, respectively, thereby establishing a stabilized epidemiological baseline.

As detailed in Supplementary Table 5, the relative performance rankings of the four evaluated predictive architectures remain remarkably consistent with the primary analysis. The Transformer model continues to demonstrate superior predictive accuracy and robust generalization capability across the interpolated datasets. Specifically, on the independent test set, the Transformer architecture achieved the lowest error metrics (MAE = 3044.484, MAPE = 0.024, and RMSE = 4209.168) alongside the highest R^2 (0.923). In contrast, the baseline algorithms, including the GRU, CNN, and SVM models, exhibited comparatively lower predictive precision.

The accompanying visual diagnostics further corroborate these quantitative findings. The temporal tracking trajectories reveal that the Transformer model exceptionally captures the underlying cyclical patterns and trend evolutions without being distorted by historical extremes. Furthermore, the correlation scatter plots enhanced with marginal distributions and statistical confidence intervals illustrate that the predictions generated by the Transformer tightly cluster along the ideal linear fit line. The notably narrow 95% confidence bounds and the high degree of distributional overlap between the training and testing sets confirm the structural stability of the model (**Supplementary Figure 7-9**).

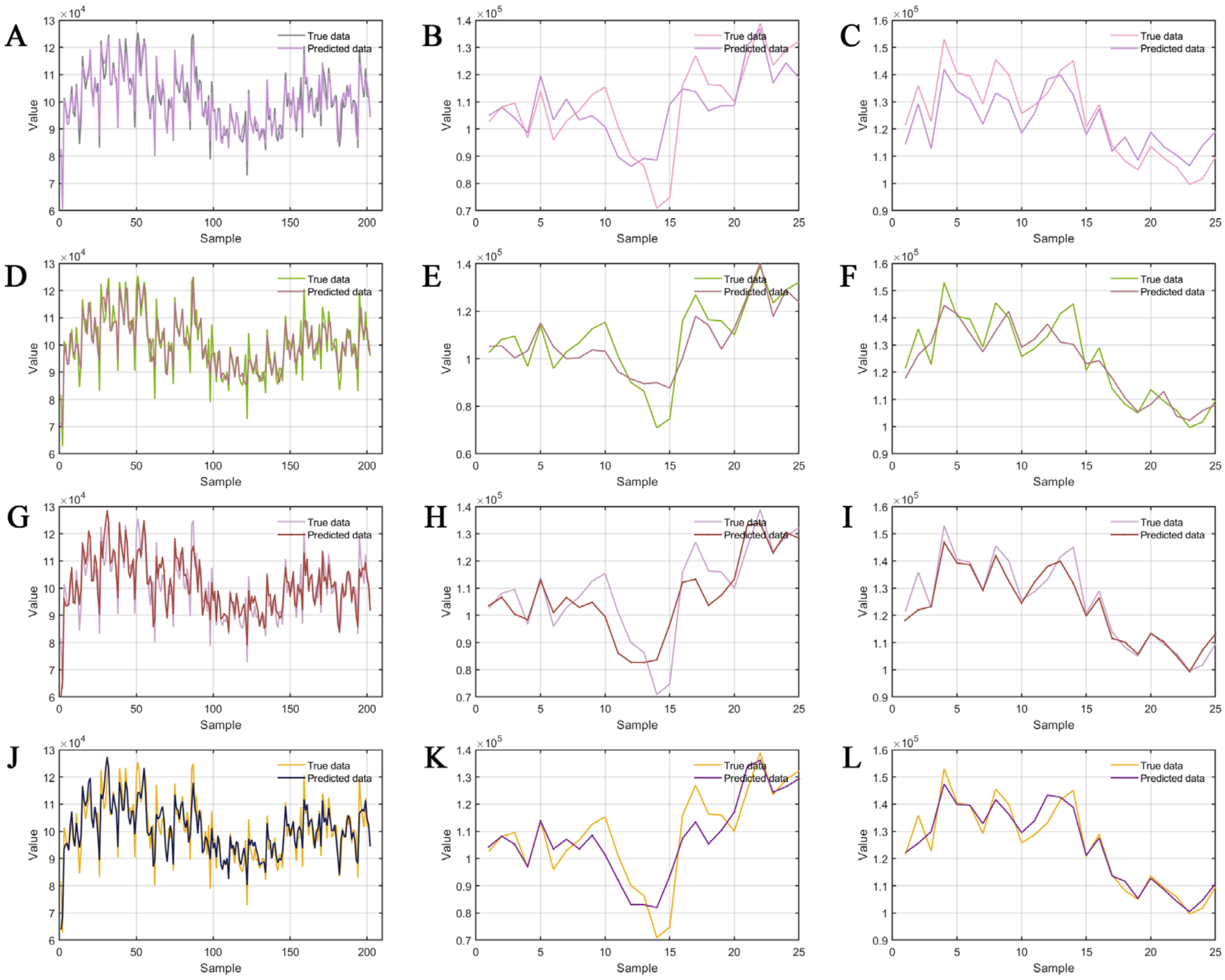

Supplementary Figure 7. Temporal tracking trajectories of the true versus predicted data for all evaluated models based on the interpolated dataset. Notes: Panels A through C represent the GRU model. Panels D through F represent the CNN model. Panels G through I represent the SVM model. Panels J through L represent the Transformer model. The training sets correspond to panels A, D, G, and J. The validation sets correspond to panels B, E, H, and K. The test sets correspond to panels C, F, I, and L.

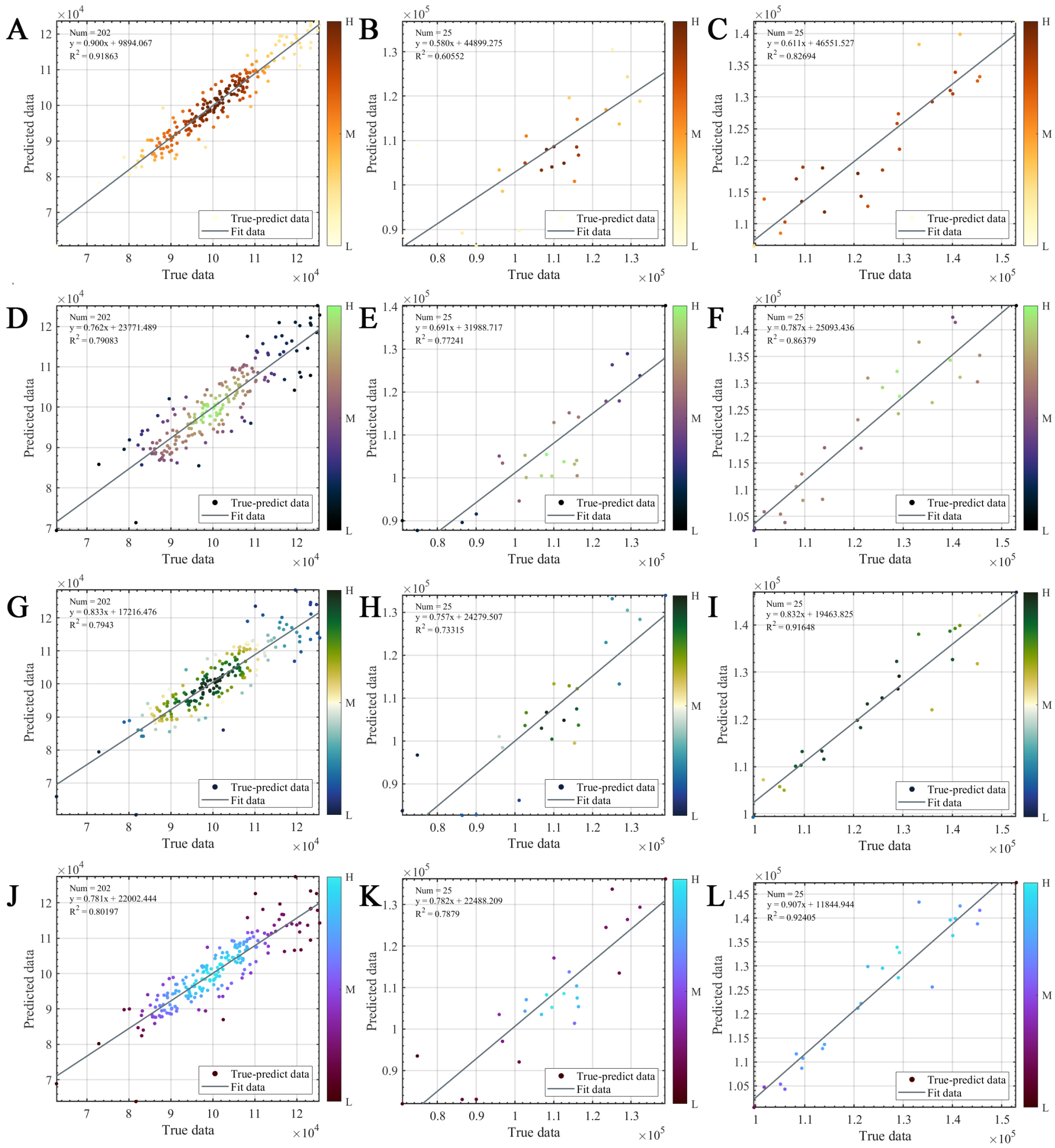

Supplementary Figure 8. Scatter plots illustrating the linear correlation and prediction density between the observed and forecasted values in the sensitivity analysis. Notes: GRU: A–C; CNN: D–F; SVM: G–I; Transformer: J–L. Training sets: A, D, G, J, validation sets: B, E, H, K, test sets: C, F, I, L.

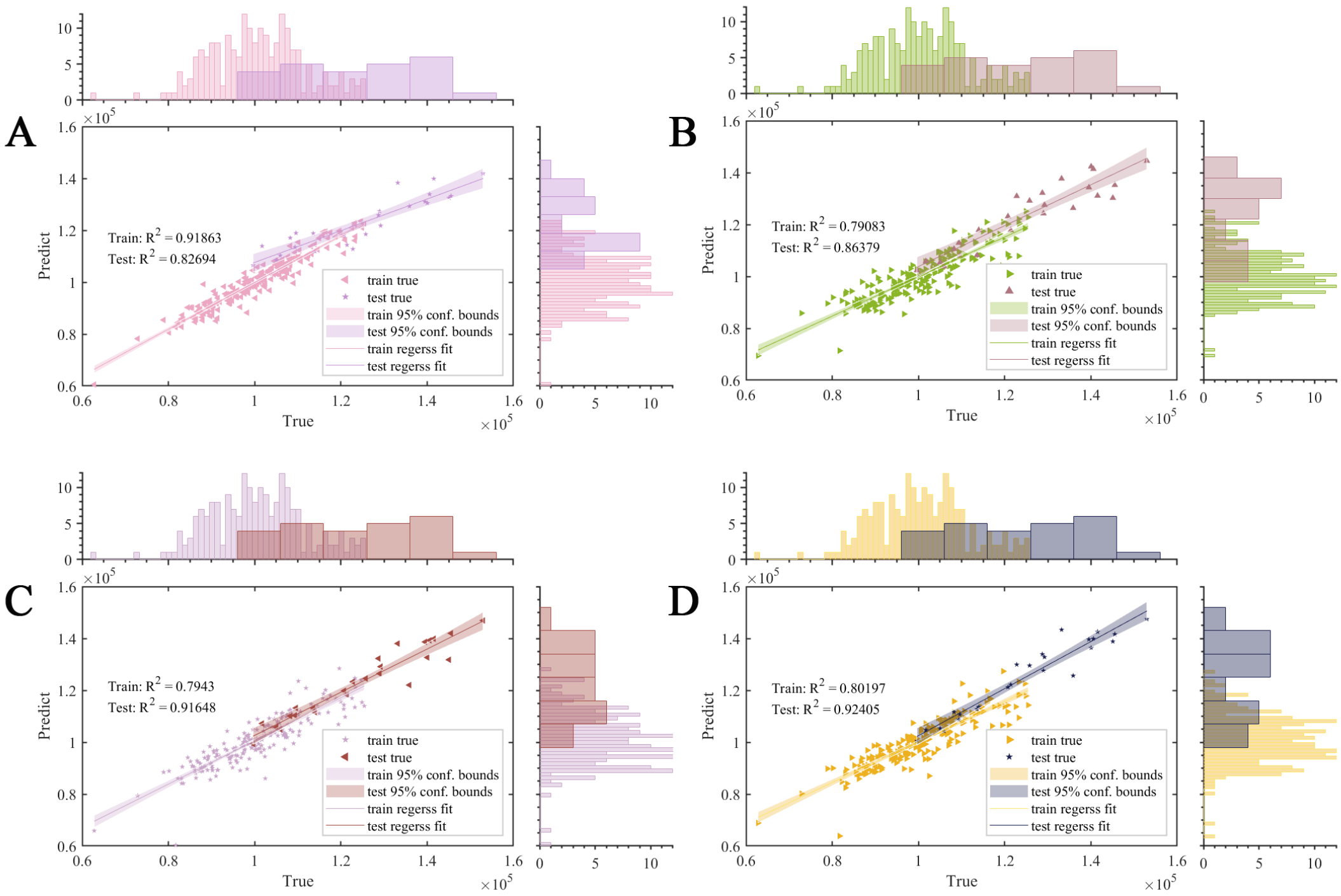

Supplementary Figure 9. Advanced scatter diagnostics featuring marginal histograms and 95% confidence bounds for the training and testing sets. Notes: Panels A through D display the fitting results of the GRU, CNN, SVM, and Transformer models, respectively.

Supplementary Table 5. Sensitivity analysis of comparative goodness-of-fit metrics across training, validation, and test sets

Models	Training set fitting power				Validation set fitting power				Test set fitting power			
	MAE	MAPE	RMSE	R^2	MAE	MAPE	RMSE	R^2	MAE	MAPE	RMSE	R^2
GRU	2340.022	0.024	3113.262	0.918	7666.003	0.078	10420.570	0.602	6790.504	0.055	7565.995	0.753
CNN	3697.359	0.038	4990.116	0.789	6529.364	0.065	8183.718	0.754	4799.169	0.037	5931.162	0.848
SVM	3795.965	0.038	4972.341	0.791	6890.876	0.068	8819.186	0.715	3256.374	0.025	4848.762	0.898
Transformer	3578.121	0.036	4846.441	0.801	6091.360	0.060	7712.846	0.782	3044.484	0.024	4209.168	0.923

The prediction results of the models

Supplementary Tables 6-9 present the 24-month forecasts of reported Hepatis B cases in mainland China produced by the GRU, CNN, SVM, and Transformer models, respectively, together with the corresponding 95%, 65%, and 35% prediction intervals.

Supplementary Table 6. GRU Prediction

Time	Predicted data	95%CI-upper	65%CI-upper	35%CI-upper	95%CI-lower	65%CI-lower	35%CI-lower
2026-01	99305.40	116119.61	109807.94	105863.15	89689.51	95606.70	99157.01
2026-02	97992.63	121221.36	108620.67	105579.13	89936.89	96019.99	99496.04
2026-03	100378.88	125272.58	110481.83	107068.58	86589.07	96259.95	99673.20
2026-04	107055.97	131427.37	116399.24	112931.21	92701.04	102527.12	105995.15
2026-05	106916.54	129816.42	115943.73	111833.30	93336.37	103098.64	106181.46
2026-06	107889.36	131928.17	116763.65	113514.11	92392.10	102682.31	107556.62
2026-07	110335.82	132689.75	120890.38	116273.24	96265.63	104473.88	109091.02
2026-08	114651.71	133455.80	122826.81	118840.94	100683.08	109097.69	112640.69
2026-09	110768.29	128108.25	119034.42	114909.96	97999.65	105011.24	108310.81
2026-10	107010.15	127269.49	115697.65	112175.78	91547.71	100603.94	105132.05
2026-11	107676.43	127432.75	117555.00	113439.27	67343.10	98622.65	104384.67
2026-12	112251.16	133054.44	123508.45	118735.45	73391.97	102825.46	110780.46
2027-01	108712.55	127139.41	117967.11	113380.96	71341.23	98858.14	104973.01
2027-02	107578.54	132113.95	119190.05	114343.59	69917.68	99804.20	105458.41
2027-03	112898.32	135926.89	123983.28	118409.59	74616.33	103281.01	109650.94
2027-04	117935.61	141754.29	130254.98	124947.60	78065.78	105487.22	113448.29
2027-05	116537.84	138609.26	127728.26	121381.02	76043.55	102339.28	108686.53
2027-06	116476.50	140738.11	129069.34	122786.16	75213.47	103936.60	111117.39
2027-07	118325.29	146661.30	131505.92	124875.44	78462.07	105931.21	113508.90
2027-08	119430.98	146075.38	132830.61	126208.23	79851.54	106341.07	114855.57
2027-09	115521.09	143990.46	130427.96	123646.70	75209.18	101365.44	110084.20
2027-10	110078.87	143576.16	128674.40	121796.67	65628.49	96578.30	105748.62
2027-11	109877.63	140924.67	126774.99	120244.37	63645.67	97387.20	106094.70
2027-12	109050.36	143722.33	129645.11	121433.39	62778.27	95625.14	105009.95

Supplementary Table 7. CNN Prediction

Time	Predicted data	95%CI-upper	65%CI-upper	35%CI-upper	95%CI-lower	65%CI-lower	35%CI-lower
2026-01	108545.37	127278.65	118365.65	113504.01	98919.09	104185.87	107426.96
2026-02	112354.00	126827.23	119287.65	115319.45	101033.91	106192.58	108970.32
2026-03	114228.88	128887.73	119937.52	116357.43	98904.52	107854.73	110987.30
2026-04	119057.84	146072.91	125974.41	122427.63	104102.53	112969.52	115925.17
2026-05	119986.53	142525.55	128499.55	124343.70	107200.83	113434.60	117070.97
2026-06	120636.14	148118.89	130644.16	126275.48	105056.16	113169.43	117538.11
2026-07	126618.69	152931.03	136918.64	131375.89	109820.75	117826.95	122137.98

2026-08	125306.30	150716.50	137101.38	132957.64	108095.24	115790.74	123486.25
2026-09	128821.30	154453.86	141075.42	135442.39	109389.65	119247.45	124176.34
2026-10	127990.82	148556.94	137974.52	133271.22	110342.64	118573.41	123276.71
2026-11	130057.33	151450.25	140755.55	135408.20	89064.50	119366.15	124713.50
2026-12	137387.78	160038.08	147893.98	142689.38	98450.20	127075.55	133147.59
2027-01	141410.34	164636.55	153979.84	147763.42	101584.34	129114.18	136218.66
2027-02	139205.16	162724.94	151166.44	144561.58	103281.22	128875.05	134654.30
2027-03	135344.61	161325.05	150682.28	145360.91	99242.29	124962.29	132057.47
2027-04	136723.84	161067.83	151783.86	145875.88	102831.98	124775.93	132371.91
2027-05	128905.30	156995.97	146606.23	139994.59	95602.09	118270.60	126771.29
2027-06	132534.31	154401.95	146459.95	141694.75	98013.76	123428.16	130575.95
2027-07	138566.50	168061.42	155882.44	149324.52	105292.80	126840.24	135271.84
2027-08	140735.06	166808.42	156000.30	149695.55	106463.03	129880.65	137986.75
2027-09	127089.70	158751.47	143756.13	136758.30	90772.57	114765.13	122762.64
2027-10	121211.78	160505.19	143180.19	134517.69	87956.74	108530.18	117192.68
2027-11	115367.60	150750.27	135672.52	128133.63	78592.43	101209.07	110901.91
2027-12	116355.41	155166.36	138336.92	129361.23	79994.88	101312.17	111409.83

Supplementary Table 8. SVM Prediction

Time	Predicted data	95%CI-upper	65%CI-upper	35%CI-upper	95%CI-lower	65%CI-lower	35%CI-lower
2026-01	112928.55	128889.47	122551.24	119548.93	107206.06	111876.34	114545.06
2026-02	105454.63	116476.14	110550.19	107916.44	95076.88	100344.39	102978.15
2026-03	121533.95	133754.37	127244.57	123664.18	111295.56	116828.89	119107.32
2026-04	118602.50	131873.81	124672.65	121586.44	109584.50	114042.37	116785.66
2026-05	124360.27	140885.47	132048.12	128432.84	113570.03	120398.89	123210.77
2026-06	129545.42	143537.94	138261.93	133645.43	119795.91	125401.67	128369.42
2026-07	137007.78	154298.69	145662.86	141344.94	123641.49	130118.36	133572.70
2026-08	135272.87	153961.72	146371.96	142353.85	122709.77	129853.07	134317.63
2026-09	137214.51	155370.47	148272.34	143934.59	127372.28	133287.39	136442.12
2026-10	126211.80	144247.96	136845.30	133143.97	111861.34	121114.66	125741.32
2026-11	124225.34	142410.04	134480.40	130515.57	83730.64	118621.10	122585.92
2026-12	130293.17	148254.79	140645.33	136840.60	90422.91	123143.58	128470.20
2027-01	135725.85	165800.39	148653.33	144366.57	101498.93	129791.57	135793.04
2027-02	127466.29	151308.10	140542.79	135160.13	89407.53	118115.04	124394.81
2027-03	132667.17	155829.37	144589.42	140266.36	92712.75	122109.53	130755.64
2027-04	123336.16	151943.94	139600.25	133428.41	88462.12	116676.26	122848.11
2027-05	128655.24	156783.05	142804.57	137213.18	95277.74	118575.20	125098.50
2027-06	126995.12	152154.48	140232.79	134271.95	91694.50	117240.97	124053.36
2027-07	128474.14	158351.12	144836.90	138079.79	92710.60	116843.15	124565.56
2027-08	130018.06	158776.44	146129.40	139805.88	95541.23	119931.96	128062.20
2027-09	118551.52	144801.55	135167.06	129911.88	79111.81	108891.16	117649.79
2027-10	112609.56	139622.47	128975.85	123243.05	77380.68	101949.81	112596.43

2027-11	117175.41	145469.55	134674.73	129277.31	81600.16	106788.09	116683.35
2027-12	115148.99	144420.78	131272.48	125637.49	80557.61	104036.72	112489.19

Supplementary Table 9. Transformer Prediction

Time	Predicted data	95%CI-upper	65%CI-upper	35%CI-upper	95%CI-lower	65%CI-lower	35%CI-lower
2026-01	111216.95	124247.97	118548.67	115414.05	103730.49	108289.93	110569.65
2026-02	107532.99	122294.02	115691.85	112720.87	99186.41	104468.15	107109.02
2026-03	118909.56	132873.31	125675.50	122476.47	108080.84	114079.02	116878.17
2026-04	119987.20	139397.06	128425.68	125471.85	111124.67	117032.33	119986.16
2026-05	125648.78	148371.56	133158.89	129831.12	114143.05	121273.99	124126.36
2026-06	126461.77	147188.12	136623.34	132397.43	112324.35	120247.93	124473.84
2026-07	130490.45	149405.41	141246.81	136523.40	118059.20	125788.40	129223.60
2026-08	134137.98	152402.70	145081.49	141205.56	121825.90	129577.76	133453.69
2026-09	133852.39	151767.05	144411.06	139997.46	119400.68	128227.87	132641.47
2026-10	129250.11	147799.14	139615.90	135524.28	112508.90	122226.51	126829.58
2026-11	132446.14	151414.64	143198.74	138717.34	94650.25	124526.25	130501.44
2026-12	131438.64	149389.53	142259.40	138694.34	93774.52	123008.05	130138.18
2027-01	132712.32	153203.12	142073.03	138893.01	93577.63	123787.88	130942.94
2027-02	125344.82	149036.39	137584.55	132299.08	86491.70	114680.86	121728.15
2027-03	131053.79	155307.76	144107.49	138938.13	93275.49	121706.95	127737.86
2027-04	127911.46	154491.09	143045.69	136882.79	91101.19	118394.06	125437.39
2027-05	129489.59	154926.29	143434.73	136047.29	92543.52	118809.95	125376.56
2027-06	124871.54	148757.66	138050.35	132284.87	86161.05	114988.44	122401.19
2027-07	125760.97	153301.15	141912.32	135268.84	85917.25	115338.39	122930.94
2027-08	128981.43	153202.89	143347.63	138420.01	89143.72	119530.76	127743.48
2027-09	125214.88	150118.21	140566.70	135356.79	84125.97	116253.77	124068.64
2027-10	116553.54	144657.55	133442.52	126900.42	80171.11	106339.52	113816.21
2027-11	120038.78	147189.48	135385.31	129029.21	81812.52	108144.91	116317.03
2027-12	112618.60	143077.34	129476.85	121705.14	75074.87	101304.40	109076.11
